# Resistance screening and *in silico* characterization of cloned novel RGA from multi-race resistant lentil germplasm against Fusarium wilt (*Fusarium oxysporum* f. sp. *lentis)*

**DOI:** 10.3389/fpls.2023.1147220

**Published:** 2023-04-21

**Authors:** K. Nishmitha, Rakesh Singh, Sunil C. Dubey, Jameel Akthar, Kuldeep Tripathi, Deeba Kamil

**Affiliations:** ^1^ Division of Plant Pathology, ICAR-Indian Agricultural Research Institute, New Delhi, India; ^2^ Division of Genomic Resources, ICAR-National Bureau of Plant Genetic Resources, New Delhi, India; ^3^ Indian Council of Agricultural Research, New Delhi, India; ^4^ Division of Plant Quarantine, ICAR- National Bureau of Plant Genetic Resources, New Delhi, India; ^5^ Division of Germplasm Evaluation, ICAR-National Bureau of Plant Genetic Resources, New Delhi, India

**Keywords:** lentil, fusarium wilt, screening, resistance gene analogue, cloning, *in silico* characterization

## Abstract

Fusarium wilt caused by *Fusarium oxysporum* f. sp. *lentis* (*Fol*) is the most devastating disease of lentil present worldwide. Identification of multi-race fusarium wilt resistance genes and their incorporation into existing cultivars will help to reduce yield losses. In the present study, 100 lentil germplasms belonging to seven lentil species were screened against seven prevalent races of *Fol*, and accessions IC201561 (*Lens culinaris* subsp. *culinaris)*, EC714243 (*L. c*. subsp. *odemensis)*, and EC718238 (*L. nigricans)* were identified as resistant. The typical R gene codes for the nucleotide-binding site and leucine-rich repeats (NBS-LRR) at the C terminal are linked to either the Toll/interleukin 1-like receptor (TIR) or coiled coil (CC) at the N terminal. In the present study, degenerate primers, designed from the NBS region amplifying the P-loop to the GLPLA motif, isolated forty-five resistance gene analogues (RGAs) from identified resistant accessions. The sequence alignment identified both classes of RGAs, TIR and non-TIR, based on the presence of aspartate (D) and tryptophan (W) at the end of the kinase motif, respectively. The phylogenetic analysis grouped the RGAs into six classes, from LRGA1 to LRGA6, which determined the diversity of the RGAs present in the host. Grouping of the RGAs identified from *Lens nigricans*, LnRGA 2, 9, 13 with I2 revealed the structural similarity with the fusarium resistance gene. The similarity index ranged from 27.85% to 86.98% among the RGAs and from 26.83% to 49.41% among the known R genes, I2, Gpa2, M, and L6. The active binding sites present along the conserved motifs grouped the RGAs into 13 groups. ADP/ATP, being the potential ligand, determines the ATP binding and ATP hydrolysis activity of the RGAs. The isolated RGAs can be used to develop markers linked to the functional R gene. Furthermore, expression analysis and full-length gene isolation pave the path to identifying the molecular mechanism involved in resistance.

## Introduction

Lentil (*Lens culinaris* Medikus subsp. *culinaris*) is one of the most important cool-season legume food crops grown after chickpea. It is an annual, self-pollinating diploid (2n= 14) crop with a genome size of approximately 4Gb (Arumuganathan and Earle, 1991). It is the oldest crop that originated in Turkey and is now cultivated in almost all parts of the world. It is abundantly grown in North America, Africa, the Middle East, and Asia. Globally, lentil is grown in an area of 6.1 million ha and produces about 6.33MT of yield. India has the largest area under cultivation at approximately 1.51Mha and is placed second in global production after Canada, producing 1.096 million metric tons (FAO, 2018). Lentil is a rich source of protein (24-26%) and fiber. It is also a source of micronutrients such as calcium, phosphorus, and iron and essential amino acids such as lysine (Duran et al., 2004). Lentil is abundantly grown in the rabi season in the northern states of India, such as Delhi, Uttar Pradesh, Madhya Pradesh, West Bengal, Rajasthan, Punjab, and Haryana. It is also grown as a rotational rain-fed crop depending on the previous season’s residual moisture. It is considered a valuable crop, as it consumes a minimum input yet fixes atmospheric nitrogen, enhances soil fertility, and generates means of livelihood for small-scale farmers (Kumar et al., 2010). However, biotic and abiotic stresses have a significant impact on its yield. Fusarium wilt, caused by *Fusarium oxysporum* f. sp. *lentis* Vasudeva and Srinivasan, is one of the major constraints in lentil production globally and in India. The disease causes drying of leaves and seedling death during the seedling stage as well as partial or complete wilting during the reproductive stage (Jiskani et al., 2021). The presence of multiple races in the Indian subcontinent has made its control more challenging (Hiremani and Dubey, 2018). In India, it accounts for 100% of yield loss if it affects in the seedling stage (Khare, 1981) and 50-70% in natural conditions (Agrawal et al., 1993). Resistance breeding by identification and incorporation of single or multiple race-specific resistance genes into cultivars would considerably control the disease in a geographic area. A few resistant varieties have been released previously, but the potential of available germplasms has been less explored. Screening for resistance during different plant growth stages helps to identify late wilting during the reproductive stage and the temporal variation of resistance (Bayaa et al., 1997). Wild lentil species have been identified as a potential source of resistance that can be tailored for resistance breeding (Singh et al., 2020). Multi-location screening of germplasms identifies germplasms that are resistant to multiple races present in a geographic region (Meena et al., 2017). Conventionally, the resistance (R) gene is isolated by transposon tagging and map-based cloning, which is laborious and time-consuming. The structure of the R gene is conserved across plants with a typical NBS-LRR region at the C terminal linked to a coiled coil (CC) or Toll/interleukin 1 receptor (TIR) at the N terminal end (Nair and Thomas, 2007). A degenerate primer designed from the conserved NBS region can be used to PCR amplify resistance gene analogues (RGAs). Previously, RGAs were cloned in lentil (Yaish et al., 2004) and other legume crops such as chickpea, fava bean (Palomino et al., 2006), pigeon pea (Agbagwa et al., 2018), and common bean (Garzon et al., 2013). The isolated RGAs mainly belong to the TIR and non-TIR classes of NBS; they serve as a marker linked to the functional R gene and help in isolating the full-length R gene (Sekhwal et al., 2015). Previously, no lentil germplasm was identified as resistant to multiple races of *Fol*. Isolation and characterization of potential RGAs from resistant sources serve as a useful tool for full-length gene isolation. The present study was undertaken with the following objectives: 1. To screen 100 lentil accessions, which belong to one cultivated species and six wild species, to identify the resistance source against seven races of *Fol*; 2. to isolate the RGA from resistant accessions for further characterization and identification of potential interacting partners.

## Materials and methods

### Fungal isolates

Seven races of *Fusarium oxysporum* f. sp. *lentis* (MP-2, UP-9, RJ-8, DL-1, CH-5, UP-12, and BR-27) that were reported earlier (Hiremani and Dubey, 2018) were used in the present study. The pure culture of isolates was maintained in PDA slants and stored at 4°C for further study.

### Plant material

One hundred accessions belonging to *Lens culinaris* subsp. *culinaris* (70), *L. c*. subsp*. tomentosus* (2), *L. c* subsp. *orientalis* (7), *L. c*. subsp. *odemensis* (5), *L. lamottei* (3), *L. nigricans* (6), and *L. ervoides* (7) were collected from ICAR-NBPGR, New Delhi. The susceptible check (L-9-12) and resistant check (PL639) were collected from the Division of Genetics, ICAR-IARI, New Delhi, India.

### Screening and evaluation for resistance

The experiment was carried out in ICAR-NBPGR, New Delhi, in the periods of 2020-21 and 2021-22, as per the method described by Bayaa and Erikson (Bayaa et al., 1997). The seven races of *Fusarium oxysporum* f. sp. *lentis* were grown in double autoclaved sorghum seeds for 15 days. Fifteen grams of inoculum were mixed in pots containing 2kg of sterilized soil. Surface-sterilized seeds of 100 accessions were sown in the pots, and eight plants/pot were maintained at 24°C/22°C with their respective controls (untreated).

A few accessions showed poor germination after repeated sowing and thus were not further screened against the respective races in the study. These included accession IC73121 against race 7 (BR-27), IC95658 against race 2 (UP-9), IC361467 against race 5 (CG-5), IC384447 and IC53238 against race 6 (UP-12), EC718234 against race 1, 5, and 6 (MP-2, CG-5, UP-12), and EC718330 against race 4 and 6 (DL-1and UP-12).

The performance of disease pressure was compared with resistant and susceptible checks. Disease incidence was recorded every week until the pod-filling stage, and a scale of 1-9 was used (Bayaa and Erskine, 1990) to identify resistant accessions for further RGA isolation and characterization. The accessions showing less than 1% incidence were considered highly resistant and were given a scale of 1, with 2-10% incidence being resistant (3), 11-20% incidence being moderately resistant (5), 21-50% incidence being moderately susceptible (7), and with more than 50% incidence being susceptible (9).

### Genomic DNA isolation and PCR amplification

Genomic DNA of three accessions, IC201561, EC714243, and EC718238, showing resistance to the majority of races were isolated using the CTAB method with a slight modification (Murray and Thompson, 1980; Dubey and Singh, 2008). About 1g of leaves was weighed and ground to a fine powder in liquid nitrogen using a prechilled pestle and mortar. The powder was transferred to centrifuge tubes containing a 2% CTAB buffer preheated at 65°C. The mixture was incubated in a water bath at 65°C for one hour with occasional mixing followed by the addition of 15ml chloroform:isoamyl alcohol (24:1). The samples were centrifuged at 10,000g for 10 min, and the upper aqueous phase was carefully transferred to a fresh tube. The aqueous layer was precipitated with 0.6 volume of isopropanol and 0.1 volume of 3M sodium acetate for 1hr at -20°C and then centrifuged at 15,000g at 15min. The obtained pellet was washed with chilled 70% ethanol and centrifuged at 10,000g for 10min. The pellet was air dried until the ethanol evaporated completely and dissolved in 70µl TE (10 mM tris hydrochloric acid and 1 mM sodium EDTA, pH 8). The quality and quantity of the DNA samples were evaluated by 0.8% agarose gel electrophoresis and nanodrop and stored at -20°C. A previously designed degenerate single primer pair, from the conserved NBS region of the R gene amplifying the P-loop to the GLPLA motif, was used in the present study (**Supplementary Table 1**). The PCR reaction of 25µl was carried out in 0.2ml PCR tubes containing 10x Taq buffer (Thermo Fisher), 0.5µl of 10mM dNTPs, 10pmol of each degenerate primer, 1U of Taq polymerase (Thermo Fisher), and 100ng of the template. PCR amplification was carried out with the specific conditions of initial denaturing at 95°C for 5 min, followed by 36 cycles of denaturation at 95°C for 1min, annealing at 45°C for 1 min, and elongation at 72°C for 1 min, followed by final elongation at 72°C for 5 min and cooling at 4°C in a thermal cycler (Bio-rad). The amplified products were visualized by 1.2% agarose gel electrophoresis. The PCR product corresponding to 510 bp was eluted and purified using a QIAquick Gel Extraction kit (Qiagen, Hilden, Germany).

### Cloning and sequencing

The purified PCR product was ligated to pGEMT easy vector (Promega, Madison, Wis.) and cloned to E. coli JM109 according to manufacturer’s protocol. About 50 positive colonies from each transformation were screened using the colony PCR. Clones producing a band of ≈510bp were further proceeded for plasmid isolation and *Eco*RI (Thermo Fisher) restriction digestion. Plasmids were isolated using the Wizard Plus Plasmid Minipreparation Kit (Promega) and sequenced by the Sanger sequencing method.

### *In silico* characterization

The sequences were trimmed for vector contamination, and a similarity search was performed using the BLAST algorithm in the GenBank database. The amino acid sequences were deduced using Expasy. Multiple alignment of the obtained amino acids was carried out using CLUSTALX in the BioEdit software. The phylogenetic tree was constructed by the neighbor-joining method (Saitou and Nei, 1987) with Poisson correction in the MEGAX software along with the NBS region of the known R genes: N (U15605), L6 (U27081), M (U73916), RPP5 (AAFO8790.1), RPP4 (AAM18462.1), RPP1 (AT3444670), RPS4 (CAB50708.1), Mla (AAG37356), Pi-ta (ACY24970.1), Pi36 (ADF29629.1), Pib (BAA76282.2), I2 (AF004878), RPP13 (AAF42831), RPM1 (AQ39214), Prf (U65391), Gpa2 (AF195939), RPP8 (AAC78631.1.), and FOM-2 (AY583855.1) (Kumar et al., 2018). The confidence value was checked by bootstrapping 10,000 replicates from the original data. The probable full-length R gene was found when it was aligned to the lentil reference genome, Lens culinaris CDS Redberry, and a phylogenetic tree was constructed including the lentil R gene by the neighbor-joining method in MEGAX. The percent sequence similarity between the representative RGAs from each class and among the known R genes, L6, M, I2, and Gpa2, was determined by the DNAMAN 8 software using the Needleman and Wunsch (Global model) and PAM matrix score. Multiple expectation maximizations for motif elicitation (MEME) was used for the motif identification and characterization compared with the known R gene (Bailey et al., 2009). The active binding site of the RGAs and their potential ligands were determined using the web-based I-TASSER software (Zhang, 2008, Yang et al., 2015). The relationship between ABS was determined by constructing a phylogenetic tree, using maximum likelihood in MEGAX. The secondary structure of the RGAs with percent alpha helix, beta strands, and presence of the transmembrane helix were determined by the Phyre 2 Software (Kelley et al., 2015). The tertiary structure of the RGAs was determined using the I-TASSER software based on the C-score, TM-score, and RMSD (root mean square deviation), and the best-predicted tertiary structure was selected. The tertiary structure of the lentil R genes was determined using Phyre2.

## Results

### Screening of germplasms against *Fol* races

One hundred accessions of lentil were screened against seven races of Fusarium wilt from the seedling to pod filling stages at 7 days interval for two cropping seasons, 2020-2021 and 2021-2022, and were graded into five classes with a scale of 1-9 based on disease incidence (DI). The accessions showed varying degrees of resistance to races of *Fol* (**Supplementary Tables 2, 3**). The number of accessions exhibiting high-resistance (HR) responses were 24 (*L. culinaris* subsp. *culinaris*), 26 (*L. c*. subsp*. tomentosus*), 39 (*L. c* subsp. *orientalis*), 27 (*L. culinaris* sub sp. *odemensis*), 17 (*L. lamottei*), 39 (*L. nigricans*), and 26 (*L. ervoides*) in 2020-21 and 21 (*L. culinaris* subsp. *culinaris*), 24 (*L. c*. subsp*. tomentosus)*, 38 (*L. c* subsp. *orientalis*), 26 (*L. culinaris* sub sp. *odemensis)*, 17 (*L. lamottei*), 38 (*L. nigricans*), and 25 (*L. ervoides*) in 2021-22 against races 1 and 7, respectively. Wild species, such as *L. culinaris* sub sp. *odemensis* (EC714243), showed resistance to all the races of *Fol* in both seasons. Accessions belonging to *L. culinaris* subsp. *culinaris* (IC201693 and IC241532) were found to be susceptible to all the races of *Fol*.

**Figure 1:**
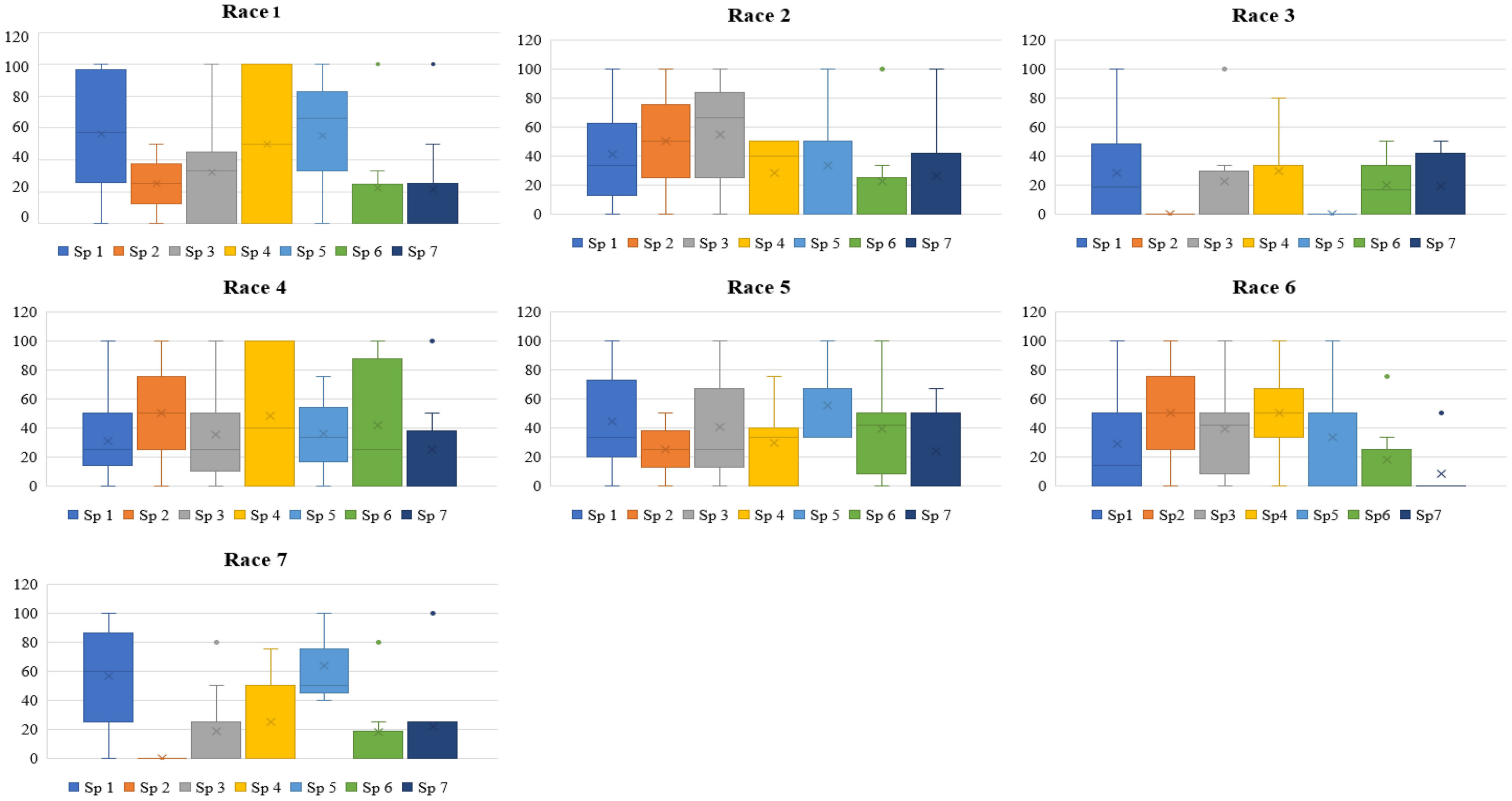
Box plot depicting disease incidence of each species of lentil against each race of *Fusarium oxysporum* f. sp. *lentis* screened during 2020-2021. Sp1: *Lens culinaris* sub sp. *culinaris*; Sp2, *L. c.* subsp*. tomentosus;* Sp3, *L. c* sub sp. *orientalis*; Sp 4, *L. c.* sub sp. *odemensis*; Sp 5, *L. lamottei*; Sp 6, *L. nigricans*; Sp 7, *L. ervoides*. Race 1: MP-2; race 2, UP-9; race 3, RJ-8; race 4, DL-1; race 5, CG-5; race 6, UP-12; race 7, BR-27.

**Figure 2:**
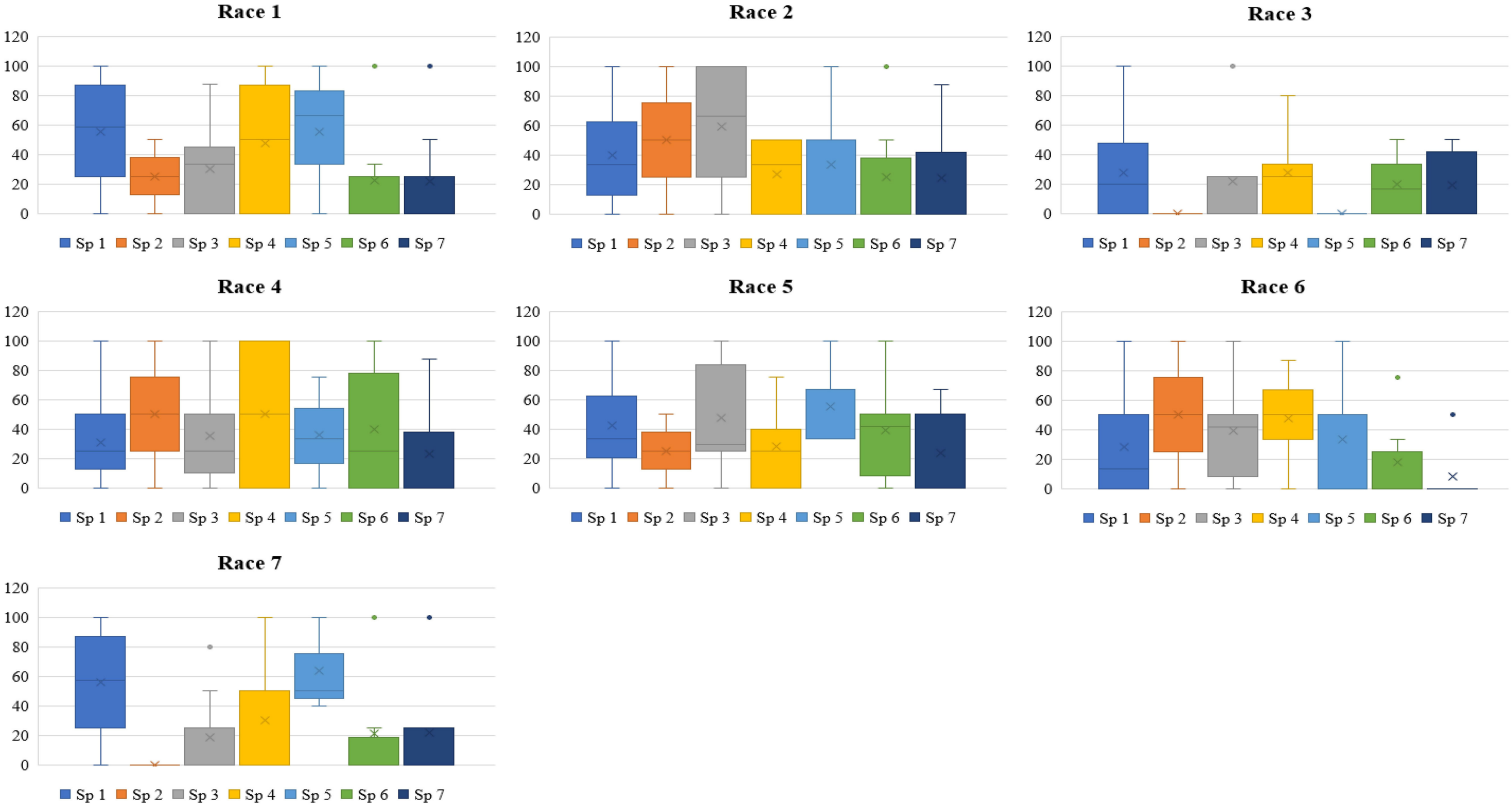
Box plot depicting disease incidence of each species of lentil against each race of *Fusarium oxysporum* f. sp. *lentis* screened during 2021-2022. Sp1, *Lens culinaris* sub sp. *culinaris*; Sp2, *L. c.* subsp*. tomentosus;* Sp 3, *L. c* sub sp. *orientalis*; Sp 4, *L. c.* sub sp. *odemensis*; Sp 5, *L. lamottei*; Sp 6, *L. nigricans*; Sp 7, *L. ervoides*. Race 1, MP-2; race 2, UP-9; race 3, RJ-8; race 4, DL-1; race 5, CG-5; race 6, UP-12; race 7, BR-27.

Accessions of *L. culinaris* subsp. *culinaris* and *L. culinaris* sub sp. *orientalis* showed the most diverse reaction, with a scale of 1-9 and mean disease incidence (DI) of 4.85-7.20 ± 0.29-0.32 and 3.00-6.67 ± 1.2-1.9, respectively, in 2020 and 4.88-7.22 ± 0.28-0.36 and 3.00-6.67 ± 1.2-1.9 in 2021, to all the races of *Fol*. All the accessions belonging to *L. c* sub sp. *tomentosa* were highly resistant to race 3 (RJ-8) and 7 (BR-27), with a mean DI of 1.00 ± 0.0 during both seasons. All the accessions of *L. lamottei* were highly resistant to race 3 (RJ-8), with a mean DI of 1.00 ± 0.0 during 2020 and 2021. In contrast, they showed moderate susceptibility to susceptible reaction, with a mean DI of 7.67 ± 0.6 to race 5 (CG-5) and race 7 (BR-27) (**Tables 1**, **2**). Variation in the mean DI between the races might be associated with the virulence of race and the differential interplay between host and race. The CV value of wild species showed large variation due to the smaller sample size and varied disease reactions within species (**Figures 1**, **2**). Variation in the disease incidence of a few accessions was observed in two seasons, which might have been due to the varied environmental conditions, such as temperature.

**Table 1:** Range of variation observed in lentil germplasm against seven races of *Fusarium oxysporum* f. sp. *lentis* in the years 2020-21.

Species	Race 1			Race 2			Race 3			Race 4			Race 5			Race 6			Race 7		
	Scale	Mean ± SE	CV	Scale	Mean ± SE	CV	Scale	Mean ± SE	CV	Scale	Mean ± SE	CV	Scale	Mean ± SE	CV	Scale	Mean ± SE	CV	Scale	Mean ± SE	CV
Sp 1	1-9	6.91 ± 0.3	39.53	1-9	6.28 ± `0.3	43.03	1-9	5.14 ± 0.3	60.07	1-9	5.69 ± 0.3	48.24	1-9	6.77 ± 0.29	35.31	1-9	4.85 ± 0.39	66.53	1-9	7.20 ± 0.32	36.62
Sp 2	1-7	4 ± 3	106.07	1-9	5.00 ± 4	113.1	1-1	1.00 ± 0	0.00	1-9	5.00 ± 4	113.14	1-7	4.00 ± 3	106.07	1-9	5.00 ± 4	113.14	1-1	1.00 ± 0.0	0.00
Sp 3	1-9	5.33 ± 1.4	64.59	1-9	6.43 ± 1.4	58.79	1-9	3.86 ± 1.3	93.99	1-9	5.57 ± 1.9	61.18	1-9	6.67 ± 1.2	44.16	1-9	5.33 ± 1.4	64.59	1-9	3.00 ± 1.31	115.47
Sp 4	1-9	5.40 ± 1.8	75.90	1-7	4.60 ± 1.4	71.44	1-9	5.00 ± 1.6	74.83	1-9	5.40 ± 1.8	75.90	1-9	5.00 ± 1.6	74.83	1-9	6.60 ± 1.47	49.79	1-9	3.80 ± 1.74	102.60
Sp 5	1-9	6.33 ± 2.6	72.93	1-9	3.67 ± 2.6	125.9	1-1	1.00 ± 0	0.00	1-9	5.67 ± 2.4	73.47	7-9	7.67 ± 0.6	15.06	1-9	3.67 ± 2.6	125.97	7-9	7.67 ± 0.67	15.06
Sp 6	1-9	3.33 ± 1.5	110.09	1-9	3.33 ± 1.5	110.0	1-7	4.00 ± 1.3	82.16	1-9	4.67 ± 1.6	87.48	1-9	5.33 ± 1.4	64.59	1-7	3.00 ± 1.2	103.28	1-9	3.33 ± 1.5	110.09
Sp 7	1-9	3 ± 1.3	115.47	1-9	3.86 ± 1.3	93.9	1-7	3.57 ± 1.2	89.80	1-9	3.33 ± 1.5	110.09	1-9	3.86 ± 1.37	93.99	1-7	2.00 ± 1	122.47	1-9	3.86 ± 1.37	93.99

Sp 1, L. c. subsp. culinaris; Sp 2, L. c. subsp. tomentosus; Sp 3, L. c subsp. orientalis; Sp 4, L. c. subsp. odemensis; Sp 5, L. lamottei; Sp 6, L. nigricans; Sp 7, L. ervoides.

**Table 2:** Range of variation observed in lentil germplasm against seven races of *Fusarium oxysporum* f. sp. *lentis* in the years 2021-22.

Species	Race 1			Race 2			Race 3			Race 4			Race 5			Race 6			Race 7		
	Scale	Mean ± SE	CV	Scale	Mean ± SE	CV	Scale	Mean ± SE	CV	Scale	Mean ± SE	CV	Scale	Mean ± SE	CV	Scale	Mean ± SE	CV	Scale	Mean ± SE	CV
Sp 1	1-9	7.22 ± 0.29	34.53	1-9	6.3 ± 0.31	43.03	1-9	5.2 ± 0.36	60.07	1-9	5.8 ± 0.32	48.24	1-9	6.71 ± 0.28	35.31	1-9	4.88 ± 0.38	66.53	1-9	7.20 ± 0.3	36.62
Sp 2	1-7	4 ± 3	106.07	1-9	5.00 ± 4	113.1	1-7	1.00 ± 0	0.00	1-9	5.00 ± 4	113.14	1-7	4.00 ± 3	106.07	1-9	5.00 ± 4	113.14	1-1	1.00 ± 0	0.00
Sp 3	1-9	5.33 ± 1.4	64.59	1-9	6.43 ± 1.4	58.79	1-9	3.86 ± 1.3	93.99	1-9	5.57 ± 1.2	61.18	1-9	6.67 ± 1.2	44.16	1-9	5.33 ± 1.4	64.59	1-9	3.00 ± 1.3	115.47
Sp 4	1-9	5.40 ± 1.8	75.90	1-7	4.60 ± 1.46	71.44	1-9	5.00 ± 1.6	74.83	1-9	5.40 ± 1.8	75.90	1-9	5.00 ± 1.6	74.83	1-9	6.60 ± 1.4	49.79	1-9	3.80 ± 1.7	102.60
Sp 5	1-9	6.33 ± 2.6	72.93	1-9	3.67 ± 2.6	125.9	7-9	1.00 ± 0	0.00	1-9	5.67 ± 2.4	73.47	7-9	7.67 ± 0.6	15.06	1-9	3.67 ± 2.6	125.97	7-9	7.67 ± 0.6	15.06
Sp 6	1-9	3.33 ± 1.4	110.09	1-9	3.33 ± 1.4	110.0	1-9	4.00 ± 1.3	82.16	1-9	4.67 ± 1.6	87.48	1-9	5.33 ± 1.4	64.59	1-7	3.00 ± 1.4	103.28	1-9	3.33 ± 1.4	110.09
Sp 7	1-9	3 ± 1.3	115.47	1-9	3.86 ± 1.3	93.9	1-9	3.57 ± 1.2	89.80	1-9	3.33 ± 1.4	110.09	1-9	3.86 ± 1.3	93.99	1-7	2.00 ± 1	122.47	1-9	3.86 ± 1.3	93.99

Sp 1, L. c. subsp. culinaris; Sp 2, L. c. subsp. tomentosus; Sp 3, L. c subsp. orientalis; Sp 4, L. c. subsp. odemensis; Sp 5, L. lamottei; Sp 6, L. nigricans; Sp 7, L. ervoides.

### Amplification and cloning of RGA

**Figure 3:**
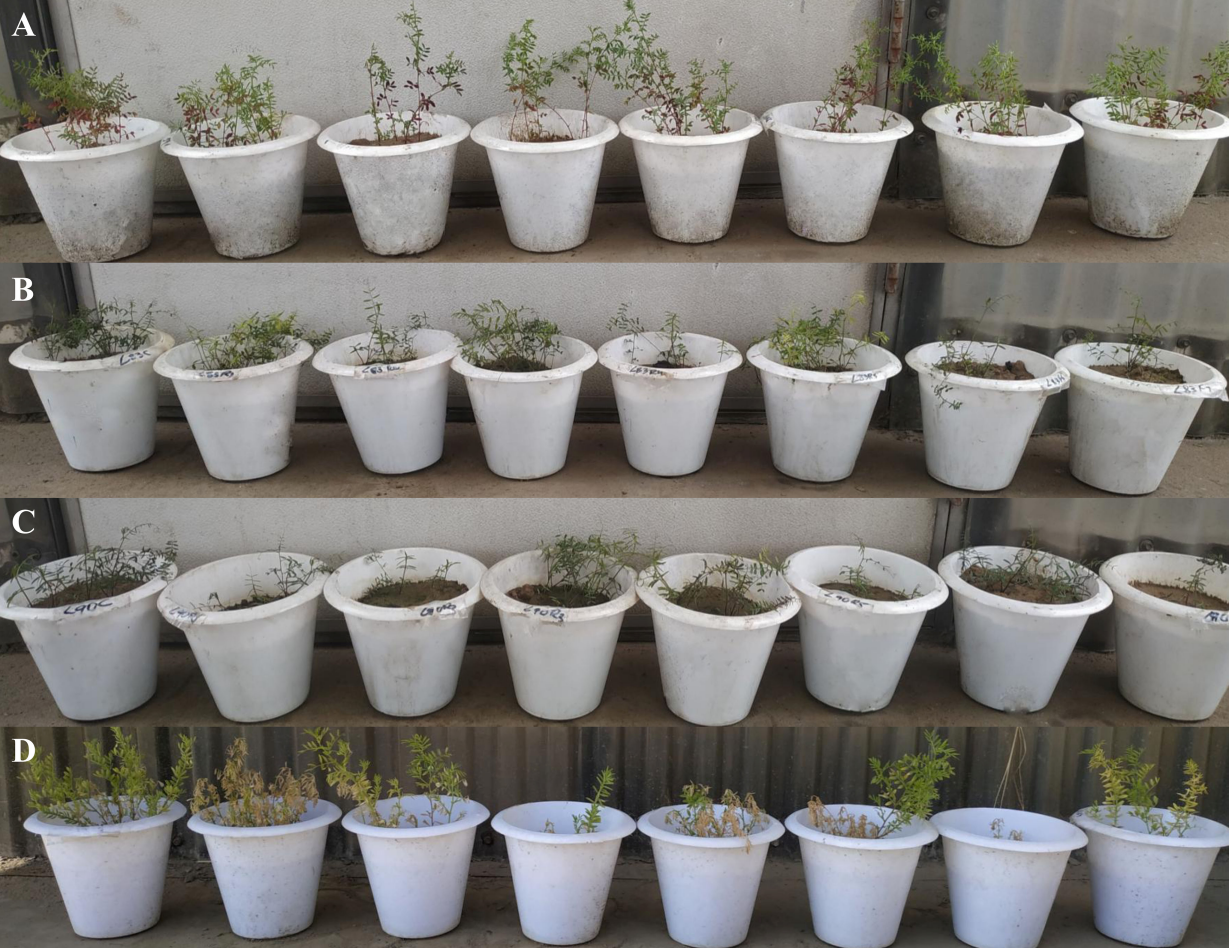
Pot evaluation of lentil accessions, **(A)** IC201561 (L65); **(B)** EC714243 (L83), **(C)** EC718238 (L90); and **(D)** L-9-12 (Susceptible check) against seven races of *Fusarium oxysporum (*f*)* sp. *lentis*. Pot in the left-hand corner is control followed by race 1 (MP-2), race 2 (UP-9), race 3 (RJ-8), race 4 (DL-1), race 5 (CG-5), race 6 (UP-12), and race 7 (BR-7).

**Figure 4:**
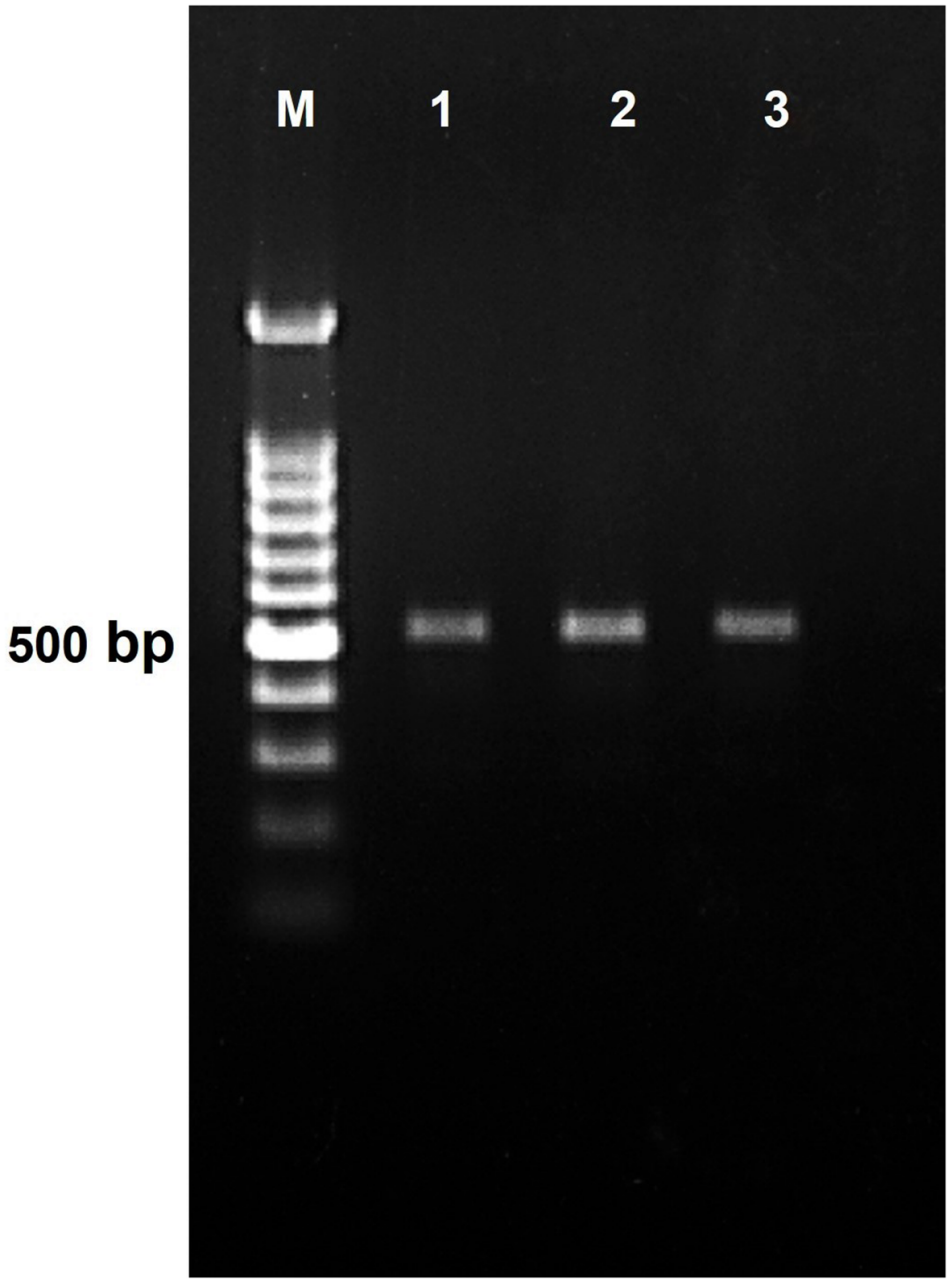
PCR amplification product generated using LRGAF and LRGAR degenerate primer on genomic DNA of lentil resistant accession. Lane 1 IC201561 (L65), Lane 2 EC714243 (L83), and Lane 3 EC718238 (L90). M represents 100 bp ladder.

After screening, the accessions with seven races of *Fol*—IC201561 (*L. culinaris*. subsp. *culinaris*), EC714243 (*L. c*. subsp. *odemensis*.), and EC718238 (*L. nigricans*)—demonstrated resistance response to *Fol* and were used for RGA isolation and characterization (**Figure 3**). The degenerate primer was designed based on the conserved region of NBS amplifying the P-loop to the GLPLA region (**Supplementary Table 1**) and used for PCR amplification of genomic DNA of selected resistant accessions, IC201561, EC714243, and EC718238. An amplicon of 510 bp in size was eluted and cloned to the pGEMT vector and *E. coli* JM109 cells (**Figure 4**). Fifty positive clones from each accession were selected for the colony PCR, and clones producing ≈510 bp further proceeded for plasmid isolation. Plasmids were isolated and restriction digested using *Eco*RI to confirm the insert and sequenced (**Supplementary Figures 1–3**). Among the 90 sequences, 45 sequences showed high similarity to the known R gene and were deposited in the NCBI database (**Supplementary Table 4**). These sequences were translated using Expasy, and the sequences showed similarity ranging from 76 to 91% with a known R gene, RUN1 and RRP13 of *Medicago truncatula* and N of *Trifolium partense*, and 84-99% similarity with a previously isolated lentil, pea, and French bean RGA (**Supplementary Table 4**).

### Multiple sequence alignment and phylogenetic analysis of RGAs

**Figure 5:**
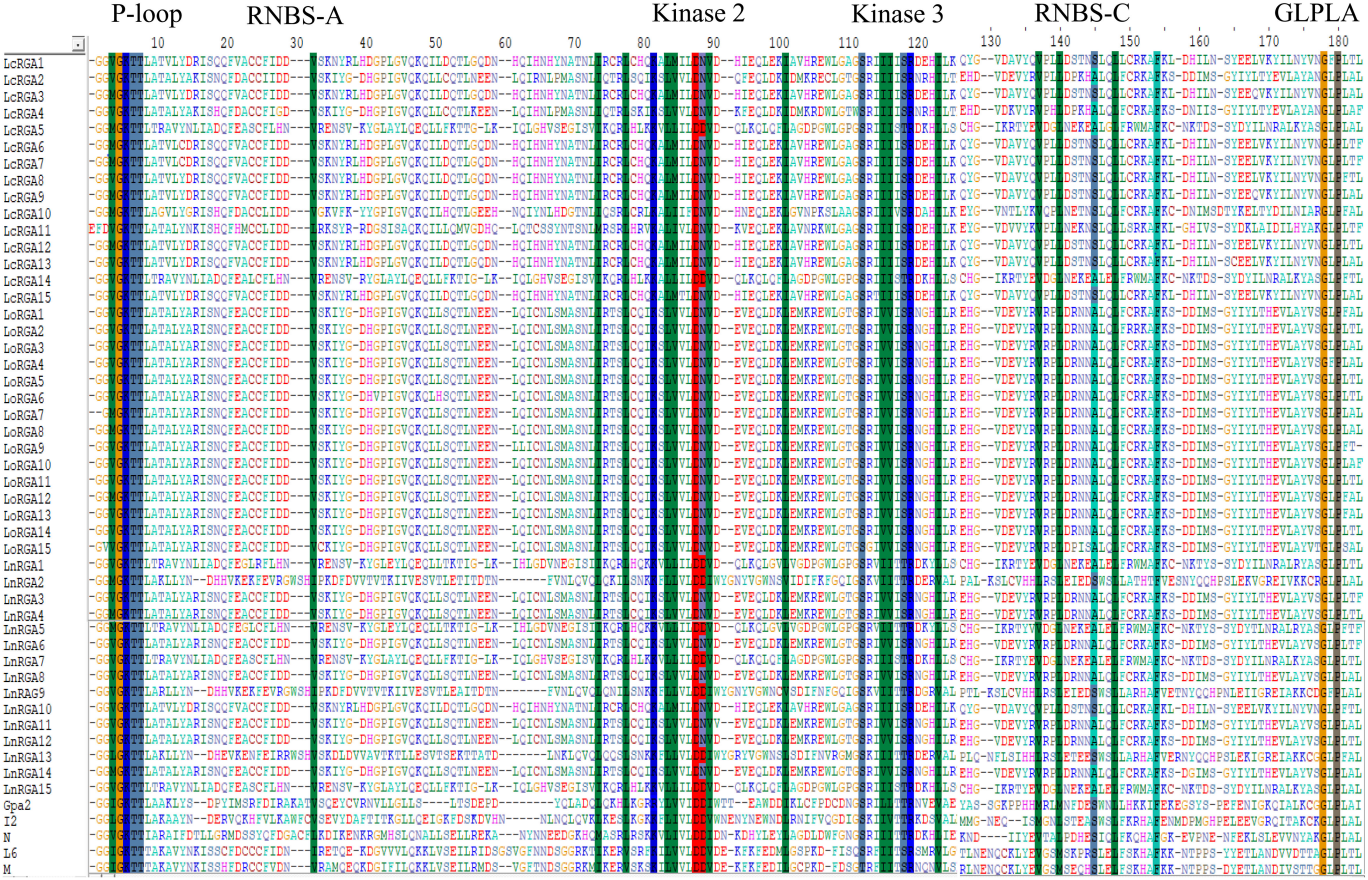
Multiple sequence alignment between P-loop and GLPLA of 45 lentil RGAs with NBS region of known R gene Gpa2, I2, N, L6, and M. The conserved motifs are indicated above the alignment. The number of amino acids is indicated above the alignment. The gaps to optimize the alignment are designated by dash (-). The alignment was constructed using CLUSTA W of Bioedit.

Multiple sequence alignment of the deduced amino acid sequences of the RGAs and known R genes, N, L6, M, I2, and Gpa2, revealed the presence of conserved motifs such as P-loop, RBNS-A, kinase 2, kinase 3, RBNS-C, and GLPLA (**Figure 5**).

**Figure 6:**
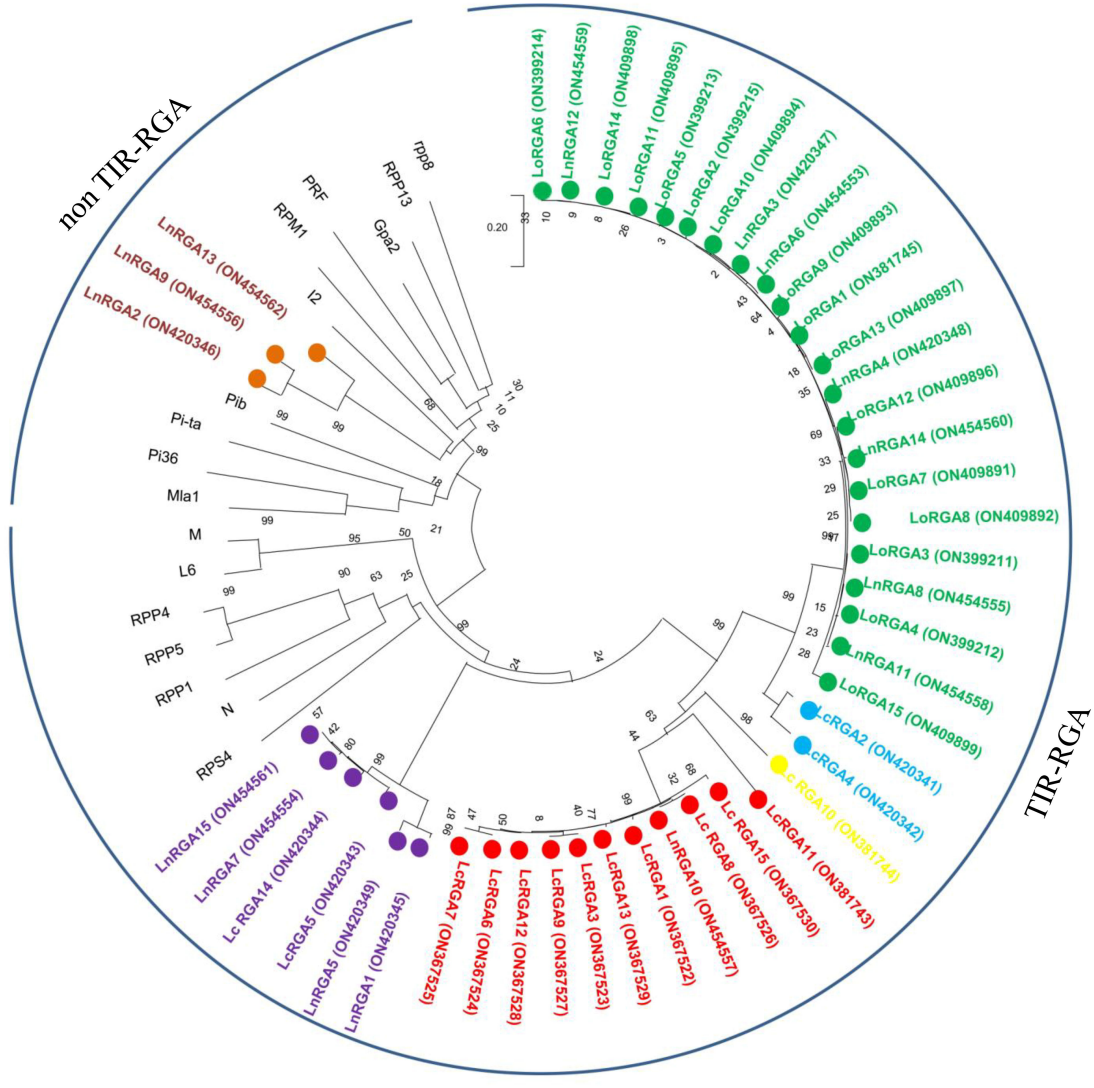
Neighbor-joining phylogenetic tree constructed based on isolated Lentil RGAs and known NBS region of R gene, N (U15605), L6 (U27081), M (U73916), RPP5 (AAFO8790.1), RPP4 (AAM18462.1), RPP1 (AT3444670), RPS4 (CAB50708.1), Mla (AAG37356), Pi-ta (ACY24970.1), Pi36 (ADF29629.1), Pib (BAA76282.2), I2 (AF004878), RPP13 (AAF42831), RPM1 (AQ39214), Prf (U65391), Gpa2 (AF195939), RPP8 (AAC78631.1.), FOM-2 (AY583855.1) at bootstrap values (10000 replicates). Numbers on the branches indicate the percentage of bootstrap replications. Green represents LRGA 1, blue represents LRGA 2, yellow represents LRGA 3, red represents LRGA 4, purple represents LRGA 5, and orange represents LRGA 6.

**Figure 7:**
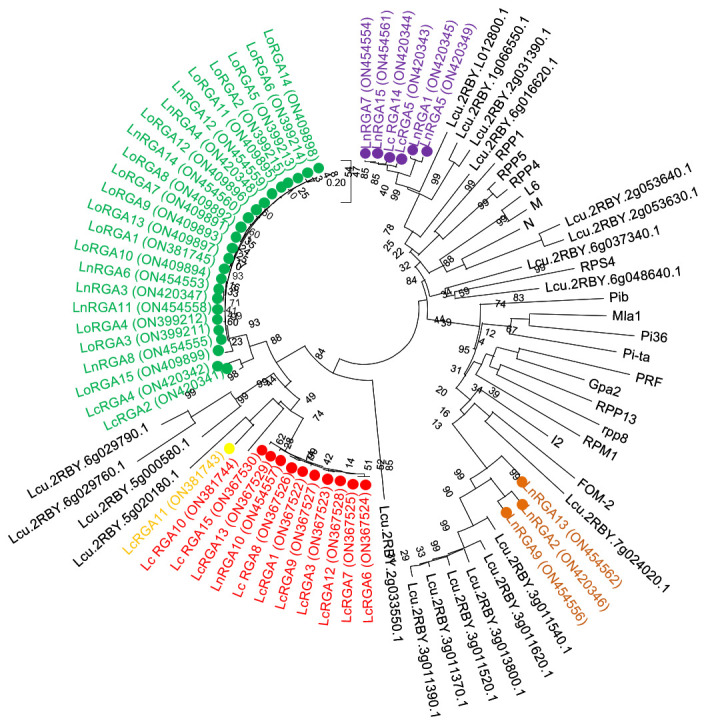
Neighbor-joining phylogenetic tree constructed based on isolated lentil RGAs, lentil R gene, and known NBS region of R genes N (U15605), L6 (U27081), M (U73916), RPP5 (AAFO8790.1), RPP4 (AAM18462.1), RPP1 (AT3444670), RPS4 (CAB50708.1), Mla (AAG37356), Pi-ta (ACY24970.1), Pi36 (ADF29629.1), Pib (BAA76282.2), I2 (AF004878), RPP13 (AAF42831), RPM1 (AQ39214), Prf (U65391), Gpa2 (AF195939), RPP8 (AAC78631.1.), and FOM-2 (AY583855.1) at bootstrap values (1000 replicates). Numbers on the branches indicate the percentage of bootstrap replications. Green represents LRGA 1, blue represents LRGA 2, yellow represents LRGA 3, red represents LRGA 4, purple represents LRGA 5, and orange represents LRGA 6.

The phylogenetic tree was constructed using the neighbor-joining method to determine the relationship between the obtained RGAs and the known R genes. The resulting tree gave rise to two branches, TIR-NBS-LRR and non-TIR-NBS-LRR. All the lentil RGAs were grouped in the TIR branch except for LnRGA2, LnRGA9, and LnRGA13, which were grouped in the non-TIR branch. All lentil TIR-RGAs and R genes L and M were clustered separately from RPP4, RPP5, RPP1, N, and RPS4. The TIR-RGAs were further classified into five classes, LRGA1-LRGA5, comprising 22, 2, 1, 11, and 6 lentil RGAs in each class. All the LoRGAs isolated from *L. c*. subsp. *odemensis* were clustered together in class LRGA1, reflecting the sequence homology among themselves. The RGAs isolated from cultivated species and wild species were grouped in LRGA 4 and LRGA 5, revealing their conserved nature. Clustering of LnRGA3, 9, and 13 into class LRGA6 along with the R gene I2 reveals their sequence homology with the fusarium wilt resistance gene. Grouping of isolated RGAs to TIR and non-TIR classes reflects the diversity of the RGAs present in the genus (**Figure 6**). The isolated RGAs were aligned with the lentil reference genome, Lens culinaris CDS Redberry. Twenty R genes from the reference genome showed high similarity with the isolated RGAs from the present study. Phylogenetic analysis revealed the clustering of lentil R genes and isolated RGA together (**Figure 7**).

The percent similarity of amino acids among the lentil RGAs and between R genes L6, M, I2, and Gpa2 was determined using DNAMAN 8 software. Amino acid similarity ranged from 27.85% (LcRGA2 and LnRGA2) to 86.98% (LnRGA1 and LcRGA5) among the RGAs. Similarity ranged from 26.83% (LnRGA13 and L6) to 49.41% (LnRGA13 and I2) when compared with the known R genes (**Table 3**).

**Table 3:** Homology matrix obtained between representative lentil RGAs and known R gene using DNAMAN 8.0.

LoRGA6	100												
LcRGA2	83.35	100											
LcRGA10	55.29	57.65	100										
LcRGA15	61.18	62.72	63.31	100									
LcRGA11	53.53	57.75	57.65	62.95	100								
LnRGA1	39.76	38.46	40.12	37.95	37.95	100							
LcRGA5	42.33	41.32	42.52	40.24	41.67	86.98	100						
LnRGA2	30.77	27.85	29.68	30.26	28.77	36.88	31.97	100					
LnRGA13	31.56	32.69	30.38	31.16	30.52	32.86	34.75	36.20	100				
I2	30.38	31.61	30.82	32.28	29.03	30.92	32.03	44.71	49.41	100			
L6	34.32	35.53	34.71	30.54	35.12	37.42	38.03	34.44	26.83	34.18	100		
M	34.91	37.95	36.59	33.54	34.12	38.79	40.37	34.44	26.99	36.48	81.98	100	
Gpa2	27.81	32.69	28.13	31.29	30.41	32.86	34.75	36.20	42.14	37.87	33.33	32.03	100

### Motif identification and characterization

**Figure 8:**
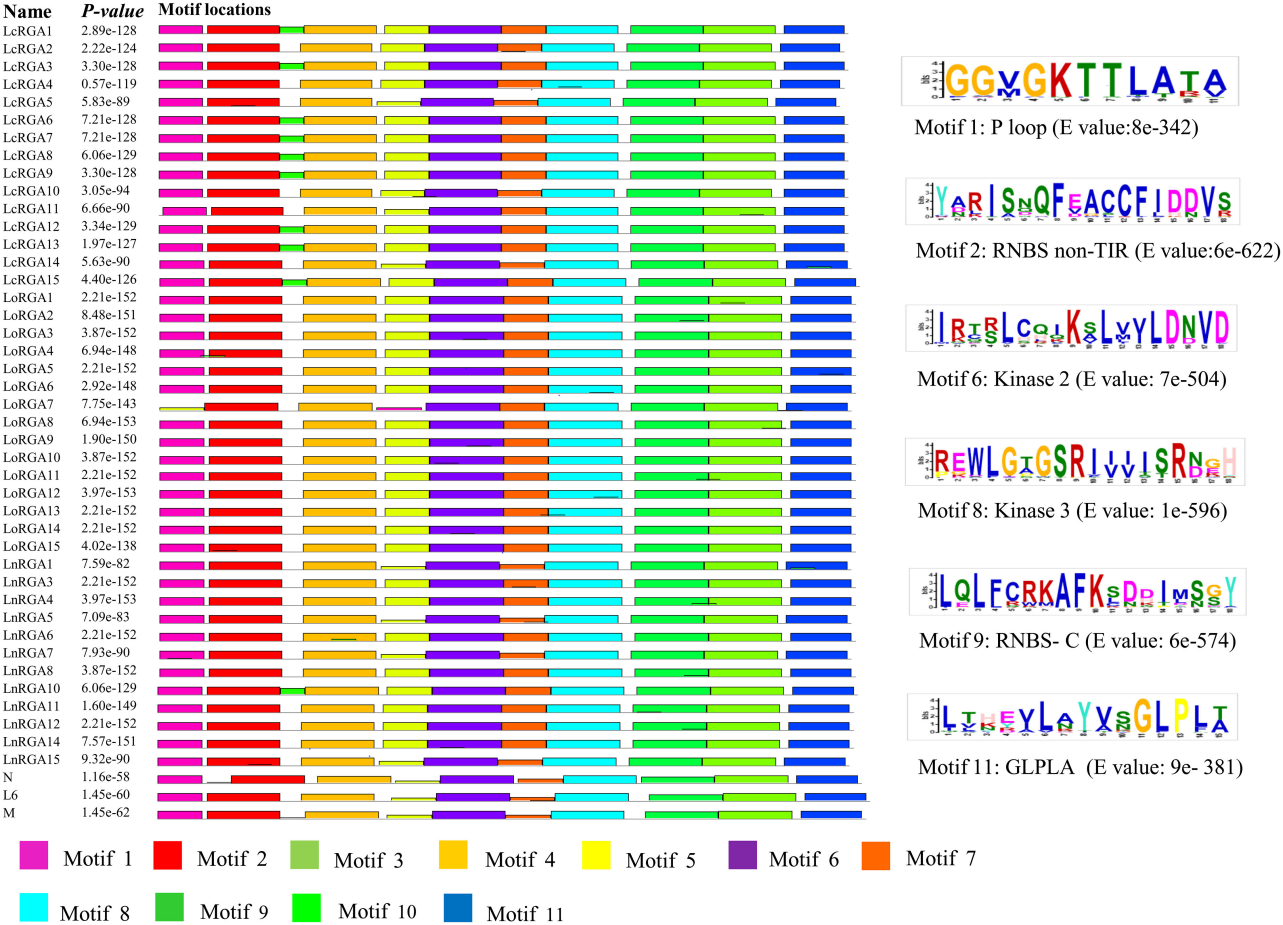
Diagrammatic representation of conserved motif of TIR RGAs within NBS domain along with R genes N, L6, and M. The solid black line represents each RGA, and their lengths with motifs are represented in colored boxes. The sequence logo of six conserved motifs along with their E value is presented on the right-hand side.

**Figure 9:**
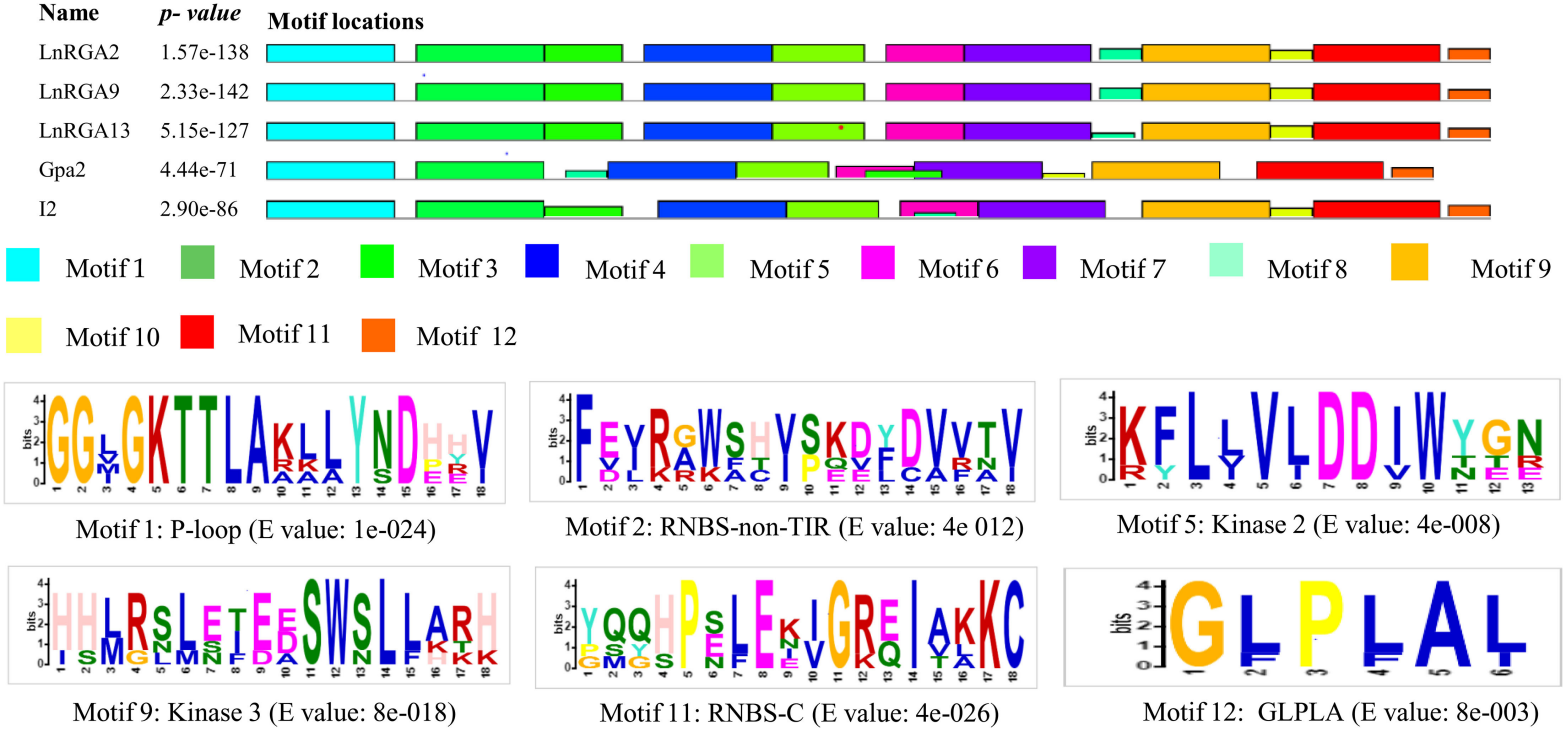
Diagrammatic representation of conserved motif of non-TIR RGAs within NBS domain along with R genes Gpa2 and I2. The solid black line represents each RGA, and their lengths with motifs are represented in colored boxes. Sequence logo of six conserved motifs along with their E value is given below.

Motifs of TIR and non-TIR RGAs were determined using the Multiple Expectation maximizations for Motif Elicitation software along with the known R genes. Eleven and twelve motifs were identified in the TIR and non-TIR groups, respectively. Six conserved motifs—P-Loop, RNBS-A, kinase 2, kinase 3, RNBS-C, and GLPLA—were found in all the RGAs. External motif, P-Loop, and GLPLA and internal motif, kinase 2, kinase 3, and RNBS-C were found conserved in both groups of RGAs. We further classified RNBS-A to TIR with amino acid sequence [(YC)(AND)(RLK)I(SA) (NQDH)QF(EVDH)(AGM)(CSL)C(FL)(ILV)(DH)(DN)(NI)(SRG)] and the non-TIR [F(CVD)(YL)(RK)(GAR)(WK)(SFA)(HTL)Y(SP)(KQE)(DVE)(YFL)(DC)(VA)(VRF)(TNA)(VI)] group due to the difference in position and composition of the motif. The presence of tryptophan (W) in the non-TIR group at the end of the kinase 2 motif distinguishes it from the TIR group with aspartate (D) amino acid. An extra motif with signature (KE)NYRLH and E-value 0E-054 was found in LcRGA 1, 3, 6, 7, 8, 9, 12, 13, and 15 and LnRGA10, all belonging to class LRGA3 of TIR-RGA (**Figures 8**, **9**).

### Prediction of active binding sites and their putative ligands

**Figure 10:**
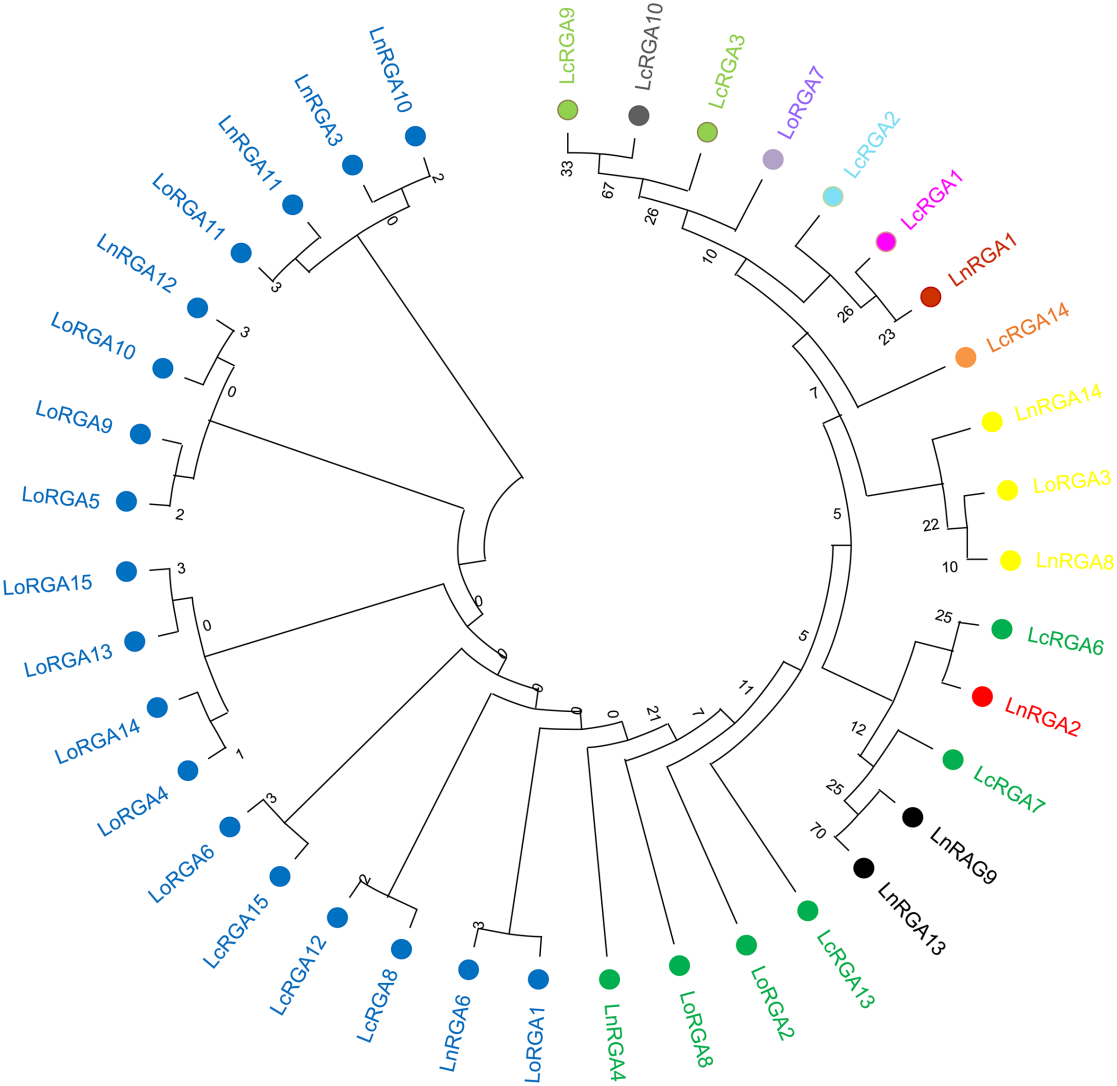
Phylogenetic tree constructed based on amino acid corresponding to active binding sites (ABS) using maximum likelihood in MEGA X with 1000 bootstrap replication. Lentil RGAs with the same color represent having a similar ABS. Blue represents GTDN, green represents GTD, black represents GTDG, red represents GTDT, yellow represents GTDL, orange represents GT, red represents L, pink represents GTS, light blue represents SN, purple represents G, light green represents GTNS, and gray represents S.

All the RGAs showed the structural analogy to know the resistance genes RPP1 and Roq1 with the molecular function of ATP binding (**Supplementary Table 5**). I-TASSER software was used to predict the active binding site of the RGAs and their respective ligands. The identified active binding sites (ABS) were present along the six conserved motifs of the nucleotide-binding site (NBS) including P-loop, RNBS-A, kinase 2, kinase 3, RNBS-C, and GLPLA. Active binding sites in the P-loop motif were largely found in 35 RGAs (10 LcRGA, 14 LoRGA, and 11 LnRGA) followed by the kinase 2 motif in 30 RGAs (5 LcRGA, 14 LoRGA, and 11 LnRGA). The amino acids that were found to be corresponding to the active binding site were glycine (G) and threonine (T) in the P-loop and aspartic acid (D) and aspergine (N) in the kinase 2 motifs; these were common in TIR and non-TIR RGAs but differed at the kinase 2 motif. It was observed that non-TIR had aspartic acid as ABS, while TIR-RGA had aspartic acid and aspergine as ABS. P-loop, kinase 2, and kinase 3 motifs were reported to have ATP/GTP binding sites in the nucleotide-binding site of the R gene. Interestingly, the aspartic acid (D) found in TIR-RNBS motif was found to be an active binding site in 17 TIR-RGAs largely isolated from *Lens culinaris* subsp. *odemensis*. LoRGA3, LnRGA8, and LnRGA14 had leucine (L) in RNBS-C, and LnRGA9 and LnRGA13 had glycine (G) in the GLPLA motif as active binding sites. ADP/ATP was found to be a potential ligand of RGAs, with the molecular function of ATP binding and ATPase activity (**Supplementary Table 6**). Previously, the involvement of RNBS-A, RNBSC, and the GLPLA motif in ATP binding and hydrolysis had not been reported. Based on amino acids corresponding to ABS, the RGAs were classified into thirteen groups (**Table 4**). Eighteen RGAs with the active binding site at 4th, 6th, 27th, and 82nd/83rd positions corresponding to G, T, D, and N amino acids were grouped in the GTDN class. Seven classes of ABS had a single lentil RGA, describing the diversity of ABS within the genus. Seven RGAs had no ABS and were considered inactive due to point mutation. Little correlation was found between ABS of RGAs and the previously constructed phylogenetic tree. The RGAs belonging to the non-TIR group had different ABS from the TIR group. The phylogenetic tree was constructed by maximum likelihood in MEGAX, aligning all the active binding sites of the RGAs with a bootstrap of 1000 replication. The tree grouped all RGAs with GTDN active binding sites together. The RGAs LnRGA9, LnRGA13 (GTDT), and LnRGA2 (GTDG) were grouped along with the GTD group, probably due to the change in single amino acids (**Figure 10**). The secondary and tertiary structure of RGAs and lentil R gene

**Table 4:** Lentil RGAs grouped based on amino acid corresponding to predicted active binding sites (ABS).

GTS	SN	GTNS	GTD	GTDN	S	GT	GTDL	G	L	GTDT	GTDG	No ABS
LcRGA1	LcRGA2	LcRGA3	LcRGA6	LcRGA8	LcRGA10	LcRGA14	LoRGA3	LoRGA7	LnRGA1	LnRGA2	LnRGA9	LcRGA4
		LcRGA9	LcRGA7	LcRGA12			LnRGA8				LnRGA13	LcRGA5
			LcRGA13	LcRGA15			LnRGA14					LcRGA11
			LoRGA2	LoRGA1								LoRGA12
			LoRGA8	LoRGA4								LnRGA5
			LnRGA4	LoRGA5								LnRGA7
				LoRGA6								LnRGA15
				LoRGA9								
				LoRGA10								
				LoRGA11								
				LoRGA13								
				LoRGA14								
				LoRGA15								
				LnRGA3								
				LnRGA6								
				LnRGA10								
				LnRGA11								
				LnRGA12								

*Each group is named based on amino acid corresponding to ABS.

The secondary structure of the RGAs was predicted using Phyre2 software and revealed the presence of alpha-helix (56%-50%), beta strands (11%-9%), and disordered sequence (12%-14%). Three RGAs, LcRGA5, LcRGA11, and LnRGA1, had the transmembrane region in the helix depicting probable interaction with the lipid bilayer (**Table 5**). The best tertiary structure of the lentil RGAs was predicted based on the C-score, TM-score, and RMSD using I-TASSER. The C-score of 45 lentil RGAs ranged from 0.31 (LcRGA14 and LoRGA2) to 0.68 (LnRGA9), and the TM-score ranged from 0.70 ± 0.10 (LnRGA11) to 0.81 ± 0.09 (LnRGA9 and LnRGA13) (**Supplementary Table 7**). The solubility of amino acids in the active binding site ranged from 1 to 4, revealing the buried nature of ABS (**Supplementary Figure 4**).

**Table 5:** Secondary structure composition of representative lentil RGAs using Phyre 2 software.

Lentil RGA	α strand	Β strand	Disorder	TM helix	confidence	identity
LoRGA6	52	10	14	–	100	39
LcRGA2	51	11	14	–	100	37
LcRGA10	54	9	13	–	100	33
LcRGA15	56	9	12	–	100	35
Lcrga11	53	9	13	9	100	32
LnRGA1	50	10	12	9	100	44
LcRGA5	51	11	14	9	100	43
LnRGA2	49	9	19	–	100	29
LnRGA13	49	9	16	–	100	32

- represents TM helix is absent.

Twenty lentil R genes showed a structural analogy to the plant NLR RPP1 tetramer in a complex with ATR1 (**Supplementary Table 8**). The tertiary structure of the lentil R gene was predicted using Phyre2 software, and the secondary structure revealed the presence of alpha-helix (27-45%), beta strands (13-19%), disordered sequence (18-23%), and transmembrane region (1-2%) (**Supplementary Figure 5**).

## Discussion

*Fusarium oxysporum* f. sp. *lentis* is a major pathogen of lentil, constraining its yield and productivity in India and worldwide. Identification of resistant germplasms through screening helps in the development of resistant cultivars and the identification of the R genes involved in the resistance mechanism. In the present study, lentil germplasms belonging to different species were screened in artificially inoculated conditions against seven race representatives of *Fol* isolates for two seasons to identify resistant germplasms and the further isolation and characterization of potential RGAs. Since Fusarium is a soil-borne pathogen, pot evaluation is considered efficient and accurate, as it takes less space, provides a uniform inoculum load and limits interaction with other soil-borne pathogens that cause synergistic effects such as *Rhizoctonia bataticola* and *Sclerotium rolfsii* (Chaudhary et al., 2010). The germplasms were screened for two consecutive years, 2020 and 2021, to increase efficiency and reduce variation (Sharma et al., 2019). The germplasms showed typical wilt symptoms, including yellowing, drooping to wilting of the plant, followed by death. In two seasons, the germplasms showed varying degrees of resistance between the races. Wild accession belonging to *Lens culinaris* sub sp. *odemensis* showed resistance to all the races of the pathogen. Multi-race resistance has been previously identified in crops such as tomato against *F. oxysporum* f. sp. *lycopersici* race 1, 2, and 3 (Reis et al., 2004) and in melon against *F. oxysporum* f. sp. *melonis* race 0, 1, and 2 (Alvarez et al., 2005). We have observed that germplasms of *L. culinaris* sub. sp. *culinaris* and *L. culinaris* sub sp. *orientalis* showed a range of reactions, from highly resistant to susceptibility, which might probably be due to the heterogeneity in the genome structure of germplasms within single species and the differential interaction of resistant genes towards particular races. Our results were in accordance with the previous reports of Brick et al. (2006) on differential resistance in the core set of the *Phaseolus vulgaris* germplasm to races 1, 2, and 4 of *Fusarium oxysporum* f. sp. *phaseoli*. The contrasting response showed by the accessions of *L. lamottei* towards races 3, 5, and 7 emphasizes the probable combinatorial interaction of multiple R genes in resistance response. Few accessions showed variation in the resistance response, as wilt is highly dependent on temperature (Ahmed et al., 2002). Higher CV has been observed in wild species due to diverse disease reactions and small sample sizes. Extensive screening has explored the potentiality of all species and subspecies of lentil against existing races of *Fol*, providing an excellent source for R gene isolation. To the best of our knowledge, this is the first report on multi-race resistance in lentil against Fusarium wilt.

In the last decade, the PCR-based approach for the isolation of genes by using a degenerate primer has been identified as a valuable tool over the conventional technique, and they have been used in crop plants to isolate resistant gene analogues that are closely associated with the R gene. In our study, accessions showing resistance responses to multiple races were used for RGA isolation and characterization. Ninety clones were isolated, and the heterogeneity within the isolated RGA amplicons was observed. Similar results were also reported in radish (Yu et al., 2018). Forty-five RGAs showed considerable sequence variation and similarity to the RUN1 and RPP13 disease resistance genes of the legume model plant, *Medicago truncatula*, and the TMV resistance gene, N of *Trifolium pratense*, predicting their role in disease resistance. Our results were in accordance with the RGA isolated from chickpea (Huettel et al., 2002). Because of the presence of conserved domain P-loop, RNBS-A, kinase 2, kinase 3, RNBS-C, and GLPLA, multiple sequence alignment revealed them to be part of the NBS region of the R gene. The amino-terminal of the typical R gene is linked to TIR (Toll/interleukin-1-like receptor) or CC (coiled coil) and is involved in defense signaling. They are differentiated based on the presence of aspartate (D) or tryptophan (W) at end of the kinase 2 motif in TIR and CC, respectively (Pan et al., 2000). We observed that forty-two isolated clones belonged to class TIR and the other three to non-TIR, which had previously not been found in Spanish lines (Yaish et al., 2004). The presence of both classes of RGAs and enhanced expression of the TIR-NBS-LRR R gene have been reported in dicots and evolved mainly through duplication and diversification during evolution (Meyers et al., 2003; Joshi and Nayak, 2013). In the present study, a significant difference in the number of lentil TIR-RGA and non-TIR RGA clones was observed. To visualize the relatedness of isolated RGAs with other known R genes, a phylogenetic tree was constructed based on the amino acid sequence. The tree differentiated the clones into two groups, TIR and non-TIR, and further into six classes, LRGA1-6. The clustering of all the RGAs isolated from *L. culinaris* subsp. *odemensis* to class LRGA1 reveals a sequence homology among the clones and could be due to tandem and segmental duplication within the sequence. The clustering of RGAs from cultivated and wild sources into the same classes reveals conserved nature of the R genes. RGAs isolated from three species were grouped into six classes, revealing the diversity of the RGAs present in the host. The diversity of RGAs might be aided by recombination and sequence exchange, resulting in haplotypic diversity (Joshi and Nayak, 2013). The grouping of the LnRGA 13 along with the I2 Fusarium-resistant gene and the sharing of 49% amino acid similarity predict the structural similarity with Fusarium wilt resistance (Sun et al., 2010). All the conserved motifs were identified in both classes of RGAs when compared with the R gene. Positional and sequence variation of the RNBS-TIR and RNBS non-TIR motif have been also observed in the Allium RGA, which is resistant to Fusarium basal rot (Rout et al., 2014).

The presence of active binding sites determines the functionality of RGAs in resistance. *In silico* characterization has identified six conserved motifs of NBS to harbor ABS, and grouping based on ABS determines the diversity of RGAs and probably the different modes of action. The nucleotide-binding site (NBS)/NB-ARC region of the R gene belongs to STAND (signal transduction ATPase with numerous domains) superfamily protein, which is involved in immunity and apoptosis (Danot et al., 2009). The biological function of all the isolated LRGAs determined using I-TASSER inferred their involvement in immunity and the involvement of LoRGA7 in intrinsic apoptotic signaling. TIR/CC-NBS-LRR requires maturation for the recognition of effector molecules and is mediated by Hsp90, an ATP-dependent chaperone, and other co-chaperones. In the closed and autoinhibited state, TIR/CC and LRR are present in close proximity and are folded back to the NBS-ARC core along with ADP. Upon recognition of the effector, conformational changes allow the exchange of ADP to ATP, resulting in an open structure and the activation of downstream defense (Takken and Goverse, 2012).

Phosphorylation of ADP has been reported as a key process in pathogen recognition (Suraby et al., 2020). The involvement of the NBS region of the I2 gene in binding to ATP at the ATP-binding sites of P-loop, kinase 2, and kinase 3, and the subsequent hydrolysis of ATP, confirmed the relatedness of NBS to the ATPase super family (Tameling et al., 2002). In the present study, ADP/ATP was found to be the potential ligand of the lentil RGAs with ATP binding and ATPase as molecular functions, and this infers its functional role in defense response. The potential solubility of ABS determined the hydrophobic nature of amino acids. We predicted the RGAs’ secondary and tertiary structure using Phyre 2 and I-TASSER. The composition of alpha-helix, beta strands, and disordered region varied between RGAs, and similar results were found in RGA isolates from watermelon resistant to Fusarium wilt (Reddy et al., 2019). The presence of a transmembrane helix deduced the role in lipid bilayer interaction (Zviling et al., 2007). The best tertiary structure of RGAs predicted with the C-score between 0.31 and 0.69 and TM-score between 0.70 ± 0.10 and 0.81 ± 0.09 was found in accordance with the previous report of Barka et al. (2020). The information generated in the present findings will be utilized for understanding the molecular-based mechanism of host–pathogen interaction.

## Conclusion

In the present study, cultivated and wild lentil species resistant to multiple races of *Fol* were identified by extensive screening. RGAs were isolated from resistant accessions that belonged to three different species using a degenerate primer. The phylogenetic analysis grouped the RGAs into six classes, determining the diversity of the RGAs present in the host. Clustering of cultivated and wild species of RGAs together revealed the conserved nature of the R gene. The molecular and biological functions revealed the ATP binding and ATP-hydrolyzing activity of the lentil RGAs, confirming their relatedness to the functional R gene. The isolated RGAs can be a useful marker associated with the R gene, and the leads obtained in the present study will be useful for expression analysis to determine its activity during pathogen interaction and will decipher the molecular mechanism involved in resistance. Multiple race-resistant genotypes can be utilized in the breeding program.

## Data availability statement

The datasets presented in this study can be found in online repositories. The names of the repository/repositories and accession number(s) can be found in the article/**Supplementary Material**.

## Author contributions

Conceptualization, KN, SD, and RS. Methodology, KN, DK, and RS. Supervision, RS, SD, JA, KT, and DK. Resources, SD and KT. software, KN and RS. Writing—original draft, KN. Writing—review and editing, RS, SD, DK, KT, and JA. All authors contributed to the article and approved the submitted version.
