# Supplementary material for: Resistance screening and in silico characterization of cloned novel RGA from multi-race resistant lentil germplasm against Fusarium wilt (Fusarium oxysporum f. sp. lentis)
Source: Front Plant Sci. 2023 Apr 21;14:1147220. doi: 10.3389/fpls.2023.1147220 (PMC10160667; doi:10.3389/fpls.2023.1147220)
Supplement: Supplementary file 1 [file DataSheet_1.docx]

Resistance screening and *in-silico* characterization of cloned novel RGA from multi-race resistant Lentil germplasm against Fusarium wilt (*Fusarium oxysporum* f. sp. *lentis)*

**Nishmitha. K^1^, Rakesh Singh^2^, Sunil C Dubey^3^, Jameel Akthar^4^,** **Kuldeep Tripathi^5^ and Deeba Kamil^1*^**

^1^Division of Plant Pathology, ICAR- Indian Agricultural Research Institute, New Delhi-110012, India

^2^Division of Genomic resources, ICAR-National Bureau of Plant Genetic Resources, New Delhi, 110012, India

^3^Indian Council of Agricultural Research, New Delhi-110001

^4^Division of Plant Quarantine, ICAR- National Bureau of Plant Genetic Resources, New Delhi, 110012, India

^5^ICAR-National Bureau of Plant Genetic Resources, New Delhi-110012

*** Corresponding author:** Deeba Kamil

email id: [deebakamil@gmail.com](mailto:deebakamil@gmail.com)

**Supplementary Table 1**. Primers used in the amplification of lentil resistance gene analogues

| **Primer name** | **Motif** | **Sequence 5’-3’** | **Amplicon** |
| --- | --- | --- | --- |
| LRGAPF | P-Loop | GGIGGIRTIGGIAARACIAC |  |
| LRGAPR | GLPLA | WTIARIGYIARIGGIARICC | 507-515 |

R=A/G; W=A/T; Y=C/T

**Supplementary Table 2**. Screening of hundred lentil germplasm against seven-race representative *Fusarium oxysporum* f. sp. *lenti*l in the year 2020-21.

| **ACCESSION** | **LINES** | **Disease incidence** | | | | | | |
| --- | --- | --- | --- | --- | --- | --- | --- | --- |
|  |  | **RACE 1 (MP-2)** | **RACE 2 (UP-9)** | **RACE3 (RJ-8)** | **RACE4 (DL-1)** | **RACE 5 (CG-5)** | **RACE 6 (UP-12)** | **RACE 7 (BR-27)** |
| **IC521438** | L1 | 11^MR^ | 12.5^MR^ | 57^S^ | 25^MS^ | 100^S^ | 14^MR^ | 12.5^MR^ |
| **IC78455** | L2 | 83^S^ | 37.5^MS^ | 14^MR^ | 16.6^MR^ | 16.1^MR^ | 0^HR^ | 100^S^ |
| **IC277173** | L3 | 87^S^ | 12.5^MR^ | 37^S^ | 0^HR^ | 62.2^S^ | 10^R^ | 40^MS^ |
| **IC544563** | L4 | 83^S^ | 25^MS^ | 28^MS^ | 0^HR^ | 80^S^ | 0^HR^ | 40^MS^ |
| **IC73121** | L5 | 0^R^ | 42.18^MS^ | 0^HR^ | 100^S^ | 75^S^ | 0^HR^ | - |
| **IC95654** | L6 | 100^S^ | 66.66^S^ | 10^R^ | 50^MS^ | 66.6^S^ | 12.5^MR^ | 83^S^ |
| **IC78549** | L7 | 0^R^ | 33.33^MS^ | 28^MS^ | 33^MS^ | 50^MS^ | 14^MR^ | 28^MS^ |
| **IC398793** | L8 | 57^S^ | 62.5^S^ | 0^HR^ | 50^MS^ | 83.3^S^ | 28^MS^ | 14^MR^ |
| **IC201582** | L9 | 66^S^ | 14.28^MR^ | 66^S^ | 14^MR^ | 40^MS^ | 14^MR^ | 12.5^MR^ |
| **IC98392** | L10 | 42^MS^ | 37.5^MS^ | 0^HR^ | 50^MS^ | 37.5^MS^ | 100^S^ | 42^MS^ |
| **IC385824** | L11 | 42^MS^ | 40^MS^ | 0^HR^ | 0^HR^ | 100^S^ | 66.6^S^ | 0^R^ |
| **IC201557** | L12 | 20^MR^ | 12.5^MR^ | 0^HR^ | 0^HR^ | 50^MS^ | 12.5^MR^ | 14^MR^ |
| **IC241533** | L13 | 20^MR^ | 0^HR^ | 0^HR^ | 25^MS^ | 14.2^MR^ | 0^HR^ | 100^S^ |
| **IC95658** | L14 | 33^MS^ | - | 0^HR^ | 25^MS^ | 28^MS^ | 0^HR^ | 50^MS^ |
| **EC199779** | L15 | 75^S^ | 50^MS^ | 0^HR^ | 20^MR^ | 28^MS^ | 14^MR^ | 14^MR^ |
| **IC260062** | L16 | 100^S^ | 42.85^MS^ | 14^MR^ | 33.3^MS^ | 62.5^S^ | 0^HR^ | 80^S^ |
| **EC33920** | L17 | 57^S^ | 0^HR^ | 25^MS^ | 0^HR^ | 20^MR^ | 0^HR^ | 42^MS^ |
| **IC201788** | L18 | 0^HR^ | 100^S^ | 50^MS^ | 0^HR^ | 60^S^ | 12.5^MR^ | 100^S^ |
| **IC14276** | L19 | 25^MS^ | 20^MR^ | 20^MR^ | 20^MR^ | 33.3^MS^ | 0^HR^ | 14^MR^ |
| **IC260010** | L20 | 100^HR^ | 0^HR^ | 42^MS^ | 14^MR^ | 14^MR^ | 42.8^MR^ | 85^S^ |
| **IC33920** | L21 | 50^MS^ | 0^HR^ | 0^HR^ | 60^S^ | 0^HR^ | 14^MR^ | 57^S^ |
| **EC11371** | L22 | 100^HR^ | 25^MS^ | 14^MR^ | 0^HR^ | 28.5^MS^ | 100^S^ | 57^S^ |
| **IC241222** | L23 | 37.5^MS^ | 25^MS^ | 100^S^ | 28.1^MS^ | 20^MR^ | 100^S^ | 0^HR^ |
| **IC396044** | L24 | 25^MS^ | 33.33^MS^ | 100^S^ | 100^S^ | 33.3^MS^ | 75^S^ | 75^S^ |
| **IC201661** | L25 | 37.5^MS^ | 75^S^ | 14.2^MR^ | 14^MR^ | 22.2^MS^ | 75^S^ | 100^S^ |
| **IC201776** | L26 | 0^HR^ | 37.5^MS^ | 0^HR^ | 12.5^MR^ | 28.1^MS^ | 28.5^MS^ | 25^MS^ |
| **IC201693** | L27 | 100^S^ | 100^S^ | 71.4^S^ | 71.4^S^ | 87.5^S^ | 57.1^S^ | 100^S^ |
| **IC201656** | L28 | 100^S^ | 12.5^MR^ | 12.5^MR^ | 100^S^ | 28.5^MS^ | 0^HR^ | 66^S^ |
| **IC98364** | L29 | 100^S^ | 100^S^ | 0^HR^ | 0^HR^ | 12.5^MR^ | 0^HR^ | 85^S^ |
| **IC201697** | L30 | 100^S^ | 0^HR^ | 0^HR^ | 0^HR^ | 12.5^MR^ | 14^MR^ | 75^S^ |
| **IC361467** | L31 | 0^HR^ | 100^S^ | 11.1^MR^ | 25^MS^ | - | 12.6^MR^ | 62^S^ |
| **EC16371** | L32 | 100^S^ | 0^HR^ | 12.5^MR^ | 16^MR^ | 42.8^MS^ | 50^MS^ | 100^S^ |
| **IC329109** | L33 | 12.5^MR^ | 0^HR^ | 20^MR^ | 14.2^MR^ | 25^MS^ | 75^S^ | 44^MS^ |
| **IC396758** | L34 | 100^S^ | 16.66^MR^ | 85^S^ | 57.1^S^ | 42.8^MS^ | 100^S^ | 0^HR^ |
| **IC241475** | L35 | 16.6^MR^ | 12.5^MR^ | 37.5^MS^ | 0^HR^ | 100^S^ | 0^HR^ | 0^HR^ |
| **IC241501** | L36 | 37.5^MS^ | 50^MS^ | 0^HR^ | 66^S^ | 100^S^ | 71.4^S^ | 100^S^ |
| **IC22651** | L37 | 100^S^ | 0^HR^ | 14.2^MR^ | 16.2^MR^ | 33.3^MS^ | 28.5^MS^ | 37^MS^ |
| **IC267088** | L38 | 100^S^ | 12.5^MR^ | 0^HR^ | 0^HR^ | 0^HR^ | 16.6^MR^ | 62^S^ |
| **IC257656** | L39 | 75^S^ | 42.85^MS^ | 28.5^MS^ | 0^HR^ | 87.5^S^ | 25^MS^ | 50^MS^ |
| **NC57762** | L40 | 37.5^MS^ | 100^S^ | 60^S^ | 14.2^MR^ | 12.5^MR^ | 0^HR^ | 100^S^ |
| **IC201555** | L41 | 100^S^ | 62.5^S^ | 37.5^MS^ | 0^HR^ | 33.3^MS^ | 0^HR^ | 25^MR^ |
| **EC28514** | L42 | 83^S^ | 100^S^ | 12.5^MR^ | 50^MS^ | 0^HR^ | 0^HR^ | 100^S^ |
| **IC22658** | L43 | 60^S^ | 0^HR^ | 83.3^S^ | 28.5^MS^ | 50^MS^ | 40^MS^ | 75^S^ |
| **EC223212A** | L44 | 100^S^ | 100^S^ | 75^S^ | 100^S^ | 100^S^ | 87.5 | 100^S^ |
| **IC241454** | L45 | 87.5^S^ | 50^MS^ | 50^MS^ | 33.3^MS^ | 22.2^MS^ | 0^HR^ | 37^MS^ |
| **EC267554** | L46 | 0^HR^ | 33.33^MS^ | 0^HR^ | 33.3^MS^ | 66.6^S^ | 100^S^ | 22^MS^ |
| **IC201748** | L47 | 75^S^ | 66.66^S^ | 28.5^MS^ | 55.5^S^ | 100^S^ | 55.5^S^ | 100^S^ |
| **IC241543** | L48 | 62.5^S^ | 16.66^MR^ | 25^MS^ | 16.3^MR^ | 25^MS^ | 83.3^S^ | 28^MS^ |
| **IC345433** | L49 | 100^S^ | 28.17^MS^ | 66.6^S^ | 57.1^S^ | 88.8^S^ | 100^S^ | 0^HR^ |
| **IC342718** | L50 | 71^S^ | 71.82^S^ | 87.5^S^ | 25^MS^ | 85.7^S^ | 50^MS^ | 55^S^ |
| **IC283540** | L51 | 62^S^ | 83.33^S^ | 28.5^MS^ | 42.2^MS^ | 100^S^ | 0^HR^ | 75^S^ |
| **IC201772** | L52 | 0^HR^ | 100^S^ | 62.5^S^ | 57.1^S^ | 25^MS^ | 75^S^ | 66^S^ |
| **IC280887** | L53 | 33^MS^ | 14.2^MR^ | 0^HR^ | 20^MR^ | 100^S^ | 20^MR^ | 0^HR^ |
| **IC201553** | L54 | 85^S^ | 50^MS^ | 28.5^MS^ | 20^MR^ | 25^MS^ | 12.5^MR^ | 87^S^ |
| **EC303712** | L55 | 100^S^ | 62.5^S^ | 0^HR^ | 14.2^MR^ | 0^HR^ | 0^HR^ | 85^S^ |
| **EC299645** | L56 | 87^S^ | 20^MR^ | 12.5^MR^ | 14.2^MR^ | 0^HR^ | 20^MR^ | 100^S^ |
| **IC201537** | L57 | 37^MS^ | 42^MS^ | 0^HR^ | 0^HR^ | 0^HR^ | 0^HR^ | 100^S^ |
| **IC384447** | L58 | 16^MR^ | 100^S^ | 0^HR^ | 66.6^S^ | 100^S^ | - | 87^S^ |
| **IC53238** | L59 | 42^MS^ | 62.5^S^ | 0^HR^ | 12.5^MR^ | 14^MR^ | - | 100^S^ |
| **IC424864** | L60 | 50^MS^ | 25^MS^ | 14.2^MR^ | 25^MS^ | 75^S^ | 0^HR^ | 100^S^ |
| **IC346268** | L61 | 25^MS^ | 12.5^MR^ | 16.6^MR^ | 33^MS^ | 25^MS^ | 0^HR^ | 0^HR^ |
| **IC616579** | L62 | 0^HR^ | 0^HR^ | 50^MS^ | 40^MS^ | 33.3^MS^ | 14^MR^ | 33^MS^ |
| **IC201694** | L63 | 0^HR^ | 25^MS^ | 20^MR^ | 28.5^MS^ | 50^MS^ | 0^HR^ | 16^MR^ |
| **IC241447** | L64 | 100^S^ | 42^MS^ | 60^S^ | 57.1^S^ | 40^MS^ | 0^HR^ | 85^S^ |
| **IC201561** | L65 | 40^MS^ | 0^HR^ | 28.5^MS^ | 14.2^MR^ | 0^HR^ | 0^HR^ | 57^S^ |
| **NC62506** | L66 | 28^MS^ | 100^S^ | 14^MR^ | 28.5^MS^ | 14^MR^ | 25^MS^ | 77^S^ |
| **IC241532** | L67 | 85^S^ | 80^S^ | 66.6^S^ | 83.3^S^ | 37.5^S^ | 100^S^ | 85^S^ |
| **EC267595A** | L68 | 28^MS^ | 25^MS^ | 25^MS^ | 50^MS^ | 12.5^MR^ | 0^HR^ | 0^HR^ |
| **IC717520** | L69 | 33^MS^ | 100^S^ | 100^S^ | 83.3^S^ | 40^MS^ | 25^MS^ | 75^S^ |
| **EC78452** | L70 | 100^S^ | 33^MS^ | 0^HR^ | 12.2^MR^ | 100^S^ | 14^MR^ | 100^S^ |
| **EC718473** | L71 | 50^MS^ | 0^HR^ | 0^HR^ | 0^HR^ | 50^MS^ | 0^HR^ | 0^HR^ |
| **EC718659** | L72 | 0^HR^ | 100^S^ | 0^HR^ | 100^S^ | 0^HR^ | 100^S^ | 0^HR^ |
| **EC718617** | L73 | 40^MS^ | 50^MS^ | 0^HR^ | 25^MS^ | 25^MS^ | 0^HR^ | 0^HR^ |
| **EC718620** | L74 | 33^MS^ | 0^HR^ | 0^HR^ | 0^HR^ | 0^HR^ | 33.3^MS^ | 0^HR^ |
| **EC718622** | L75 | 0^HR^ | 66^S^ | 25^MS^ | 75^S^ | 100^S^ | 50^MS^ | 0^HR^ |
| **EC718234** | L76 | - | 0^HR^ | 0^HR^ | 0^HR^ | - | - | 0^HR^ |
| **EC718516** | L77 | 0^HR^ | 100^S^ | 100^S^ | 20^MR^ | 25^MS^ | 100^S^ | 50^MS^ |
| **EC718623** | L78 | 50^MS^ | 100^S^ | 0^HR^ | 25^MS^ | 33.3^MS^ | 0^HR^ | 80^S^ |
| **EC718615** | L79 | 100^S^ | 66.66^S^ | 33.3^MS^ | 100^S^ | 100^S^ | 50^MS^ | 0^HR^ |
| **EC718287** | L80 | 100^S^ | 50^MS^ | 0^HR^ | 40^MS^ | 40^MS^ | 100^S^ | 75^S^ |
| **EC718694** | L81 | 50^MS^ | 0^HR^ | 33.3^MS^ | 0^HR^ | 0^HR^ | 66.6^S^ | 0^HR^ |
| **EC718312** | L82 | 100^S^ | 50^MS^ | 33.3^MS^ | 100^S^ | 33.3^MS^ | 33.3^MS^ | 50^MS^ |
| **EC714243** | L83 | 0^HR^ | 0^HR^ | 0^HR^ | 0^HR^ | 0^HR^ | 0^HR^ | 0^HR^ |
| **EC718311** | L84 | 0^HR^ | 40^MS^ | 80^S^ | 100^S^ | 75^S^ | 50^MS^ | 0^HR^ |
| **EC718252** | L85 | 100^S^ | 100^S^ | 0^HR^ | 75^S^ | 100^S^ | 100^S^ | 100^S^ |
| **EC718690** | L86 | 0^HR^ | 0^HR^ | 0^HR^ | 0^HR^ | 33.3^MS^ | 0^HR^ | 40^MS^ |
| **EC718692** | L87 | 66^S^ | 0^HR^ | 0^HR^ | 33.3^MS^ | 33.3^MS^ | 0^HR^ | 50^MS^ |
| **EC718250** | L88 | 0^HR^ | 33.33^MS^ | 0^HR^ | 0^HR^ | 50^MS^ | 0^HR^ | 0^HR^ |
| **EC718242** | L89 | 0^HR^ | 0^HR^ | 33.3^MS^ | 50^MS^ | 33.3^MS^ | 33.3^MS^ | 25^MS^ |
| **EC718238** | L90 | 33^MS^ | 0^HR^ | 0^HR^ | 0^HR^ | 0^HR^ | 0^HR^ | 0^HR^ |
| **EC718241** | L91 | 0^HR^ | 100^S^ | 33.3^MS^ | 0^HR^ | 50^MS^ | 0^HR^ | 0^HR^ |
| **EC718253** | L92 | 100^S^ | 0^HR^ | 50^MS^ | 100^S^ | 100^S^ | 75^S^ | 80^S^ |
| **EC664961** | L93 | 0^HR^ | 0^HR^ | 0^HR^ | 100^S^ | 0^HR^ | 0^HR^ | 0^HR^ |
| **EC718326** | L94 | 50^MS^ | 0^HR^ | 0^HR^ | 100^S^ | 0^HR^ | 0^HR^ | 0^HR^ |
| **EC718331** | L95 | 0^HR^ | 0^HR^ | 0^HR^ | 50^MS^ | 0^HR^ | 0^HR^ | 0^HR^ |
| **EC718329** | L96 | 0^HR^ | 50^MS^ | 50^MS^ | 0^HR^ | 0^HR^ | 0^HR^ | 25^MS^ |
| **EC718330** | L97 | 0^HR^ | 0^HR^ | 0^HR^ | - | 0^HR^ | - | 0^HR^ |
| **EC718315** | L98 | 0^HR^ | 33^MS^ | 0^HR^ | 0^HR^ | 50^MS^ | 0^HR^ | 25^MS^ |
| **EC718335** | L99 | 0^HR^ | 0^HR^ | 33.3^MS^ | 0^HR^ | 50^MS^ | 0^HR^ | 0^HR^ |
| **EC718314** | L100 | 100^S^ | 100^S^ | 50^MS^ | 0^HR^ | 66.6^S^ | 50^MS^ | 100^S^ |
| **PL639** | R check | 33^MS^ | 25^MS^ | 25^MS^ | 12^MR^ | 12^MR^ | 14^MR^ | 14^MR^ |
| **L-9-12** | S check | 100^S^ | 66^S^ | 57^S^ | 66^S^ | 80^S^ | 66^S^ | 75^S^ |

L1-L70- *L. c. subsp. culinaris;* L71-72- *L. c. subsp. tomentosus;* L73-79- *L. c subsp. orientalis;* L80- L84- *L. c. subsp. odemensis;* L85- L87- *L. lamottei;* L88-L93- *L. nigricans;* L94-L100- *L. ervoides.* HR: Highly resistant; R: resistant; MR: Moderately resistant; MS: Moderately susceptible; S: Susceptible. Dash represents accession were not screened for respective race.

**Supplementary Table 3.** Screening of lentil germplasm against seven races *Fusarium oxysporum* f. sp. *lentil* in the year 2021-22

| **ACCESSION** | **LINES** | **Disease incidence** | | | | | | | |
| --- | --- | --- | --- | --- | --- | --- | --- | --- | --- |
|  |  | **RACE 1(MP-2)** | | **RACE 2(UP-9)** | **RACE 3(RJ-8)** | **RACE 4(DL-1)** | **RACE 5(CG-5)** | **RACE 6(UP-12)** | **RACE 7 (BR-27)** |
| **IC521438** | L1 | 25^MS^ | 12.5^MR^ | | 62.5^S^ | 25^MS^ | 87^S^ | 12.5^MR^ | 12.5^MR^ |
| **IC78455** | L2 | 85^S^ | 37.5^MS^ | | 25^MS^ | 16^MR^ | 16^MR^ | 0 ^HR^ | 87^S^ |
| **IC277173** | L3 | 87^S^ | 12.5^MR^ | | 37^MS^ | 0 ^HR^ | 75^S^ | 10^R^ | 40^MS^ |
| **IC544563** | L4 | 83^S^ | 25^MS^ | | 28^MS^ | 0 ^HR^ | 80^S^ | 0 ^HR^ | 40^MS^ |
| **IC73121** | L5 | 10^R^ | 42.18^MS^ | | 0^HR^ | 100^S^ | 62.5^S^ | 0 ^HR^ | - |
| **IC95654** | L6 | 87^S^ | 75^S^ | | 0^HR^ | 50^S^ | 50^MS^ | 12.5^MR^ | 85^S^ |
| **IC78549** | L7 | 10^R^ | 25^MS^ | | 28^MS^ | 28^MS^ | 50^MS^ | 14^MR^ | 28^MS^ |
| **IC398793** | L8 | 62.5^S^ | 50^MS^ | | 0^HR^ | 50^MS^ | 83.3^S^ | 28^MS^ | 12.5^MR^ |
| **IC201582** | L9 | 66^S^ | 25^MS^ | | 66^S^ | 12.5^MR^ | 40^S^ | 5^R^ | 12.5^MR^ |
| **IC98392** | L10 | 30^MS^ | 37.5^MS^ | | 0^HR^ | 50^MS^ | 37.5^MS^ | 40^MS^ | 42^MS^ |
| **IC385824** | L11 | 50^MS^ | 40^MS^ | | 0 ^HR^ | 0 ^HR^ | 100^S^ | 66.6^S^ | 0 ^HR^ |
| **IC201557** | L12 | 12.5^MR^ | 12.5^MR^ | | 0 ^HR^ | 0 ^HR^ | 50^MS^ | 12.5^MR^ | 14^MR^ |
| **IC241533** | L13 | 20^MR^ | 10^R^ | | 0 ^HR^ | 25^MS^ | 10^R^ | 0 ^HR^ | 87^S^ |
| **IC95658** | L14 | 33^MS^ | - | | 0 ^HR^ | 25^MS^ | 25^MS^ | 0 ^HR^ | 50^MS^ |
| **EC199779** | L15 | 66^S^ | 50^MS^ | | 12.5^MR^ | 20^MR^ | 25^MS^ | 14^MR^ | 14^MR^ |
| **IC260062** | L16 | 87^S^ | 42.85^MS^ | | 14^MR^ | 33.3^MS^ | 62.5^S^ | 0 ^HR^ | 100^S^ |
| **EC33920** | L17 | 57^S^ | 10^R^ | | 25^MS^ | 0 ^HR^ | 20^MR^ | 0 ^HR^ | 50^MS^ |
| **IC201788** | L18 | 12.5^MR^ | 87^S^ | | 50^MS^ | 0 ^HR^ | 62.5^S^ | 12.5^MR^ | 100^S^ |
| **IC14276** | L19 | 25^MS^ | 20^MR^ | | 20^MR^ | 25^MS^ | 33.3^MS^ | 0 ^HR^ | 14^MR^ |
| **IC260010** | L20 | 100^S^ | 0^HR^ | | 50^MS^ | 14^MR^ | 14^MR^ | 50^MS^ | 85^S^ |
| **IC33920** | L21 | 40^MS^ | 0^HR^ | | 0 ^HR^ | 60^S^ | 0 ^HR^ | 14^MR^ | 57^S^ |
| **EC11371** | L22 | 100^S^ | 25^MS^ | | 14^MR^ | 0 ^HR^ | 28.5^MS^ | 100^S^ | 57^S^ |
| **IC241222** | L23 | 37.5^MS^ | 25^MS^ | | 87^S^ | 20^MS^ | 20 ^MR^ | 100 ^S^ | 0 ^HR^ |
| **IC396044** | L24 | 25^MS^ | 33.33^MS^ | | 100^S^ | 100 ^S^ | 33.3^MS^ | 75 ^S^ | 75 ^S^ |
| **IC201661** | L25 | 37.5^MS^ | 75^S^ | | 14.2^MR^ | 12.5 ^MR^ | 22.2 ^MS^ | 75 ^S^ | 100 ^S^ |
| **IC201776** | L26 | 0^HR^ | 37.5^MS^ | | 0^HR^ | 12.5 ^MR^ | 30 ^MS^ | 28.5^MS^ | 25 ^MS^ |
| **IC201693** | L27 | 100^S^ | 75^S^ | | 71.4^S^ | 71.4 ^S^ | 87.5 ^S^ | 75 ^S^ | 100 ^S^ |
| **IC201656** | L28 | 100^S^ | 12.5^MR^ | | 12.5^MR^ | 100^S^ | 28.5 ^MS^ | 10^R^ | 66 ^S^ |
| **IC98364** | L29 | 100^S^ | 100^S^ | | 10^R^ | 0 ^HR^ | 12.5 ^MR^ | 0 ^HR^ | 85 ^S^ |
| **IC201697** | L30 | 100^S^ | 0^HR^ | | 0 ^HR^ | 0 ^HR^ | 12.5 ^MR^ | 14 ^MR^ | 75 ^S^ |
| **IC361467** | L31 | 0^HR^ | 100^S^ | | 11.1 ^MR^ | 25^MS^ | - | 12.6 ^MR^ | 62 ^S^ |
| **EC16371** | L32 | 82.5^S^ | 0^HR^ | | 12.5 ^MR^ | 25^MS^ | 42.8 ^MS^ | 50 ^MS^ | 100 ^S^ |
| **IC329109** | L33 | 12.5^MR^ | 0^HR^ | | 20 ^MR^ | 14.2 ^MR^ | 25 ^MS^ | 75 ^S^ | 44 ^MS^ |
| **IC396758** | L34 | 100^S^ | 12.5^MR^ | | 85 ^S^ | 57.1 ^S^ | 37 ^MS^ | 100 ^S^ | 0 ^HR^ |
| **IC241475** | L35 | 16.6^MR^ | 12.5^MR^ | | 25^MS^ | 0 ^HR^ | 82.5 ^S^ | 0 ^HR^ | 0 ^HR^ |
| **IC241501** | L36 | 37.5^MS^ | 50^MS^ | | 0 ^HR^ | 66 ^S^ | 100 ^S^ | 71.4 ^S^ | 100 ^S^ |
| **IC22651** | L37 | 87^S^ | 0^HR^ | | 12.5 ^MR^ | 12.5 ^MR^ | 33.3 ^MS^ | 28.5^MS^ | 37 ^MS^ |
| **IC267088** | L38 | 100^S^ | 12.5^MR^ | | 0 ^HR^ | 0 ^HR^ | 0 ^HR^ | 12.5 ^MR^ | 50^M S^ |
| **IC257656** | L39 | 75^S^ | 42.85^MS^ | | 28.5^MS^ | 0 ^HR^ | 87.5 ^S^ | 37.5 ^MS^ | 50 ^MS^ |
| **NC57762** | L40 | 25^MS^ | 100^S^ | | 60 ^S^ | 14.2 ^MR^ | 12.5 ^MR^ | 0 ^HR^ | 87 ^S^ |
| **IC201555** | L41 | 100^S^ | 62.5^S^ | | 37.5^MS^ | 10^R^ | 33.3 ^MS^ | 0 ^HR^ | 25 ^MS^ |
| **EC28514** | L42 | 100^S^ | 87^S^ | | 12.5 ^MR^ | 50^MS^ | 0 ^HR^ | 0 ^HR^ | 100 ^S^ |
| **IC22658** | L43 | 60^S^ | 0^HR^ | | 83.3 ^S^ | 28.5^MS^ | 50 ^MS^ | 40 ^MS^ | 65^S^ |
| **EC223212A** | L44 | 100^S^ | 100^S^ | | 65^S^ | 100^S^ | 100 ^S^ | 87.5 ^S^ | 100 ^S^ |
| **IC241454** | L45 | 87.5^S^ | 33^MS^ | | 50^MS^ | 33.3 ^MS^ | 25 ^MS^ | 0 ^HR^ | 37 ^MS^ |
| **EC267554** | L46 | 0^HR^ | 33.33^MS^ | | 0^HR^ | 33.3 ^MS^ | 66.6 ^S^ | 100 ^S^ | 22 ^MS^ |
| **IC201748** | L47 | 62.5^S^ | 66.66^S^ | | 28.5 ^MS^ | 55.5^S^ | 87^S^ | 55.5^S^ | 100 ^S^ |
| **IC241543** | L48 | 62.5^S^ | 16.66^MR^ | | 25 ^MS^ | 12.5 ^MR^ | 25 ^MS^ | 83.3 ^S^ | 37.5 ^MS^ |
| **IC345433** | L49 | 87.5^S^ | 28.17^MS^ | | 66.6^S^ | 57.1^S^ | 88.8 ^S^ | 100^S^ | 12.5 ^MR^ |
| **IC342718** | L50 | 71^S^ | 71.82^S^ | | 87.5 ^S^ | 25 ^MS^ | 85.7 ^S^ | 50 ^MS^ | 55 ^S^ |
| **IC283540** | L51 | 62^S^ | 83.33^S^ | | 28.5 ^MS^ | 42.2 ^MS^ | 100 ^S^ | 10.5 ^MR^ | 75 ^S^ |
| **IC201772** | L52 | 0^HR^ | 87^S^ | | 62.5 ^S^ | 57.1 ^S^ | 25 ^MS^ | 75 ^S^ | 66 ^S^ |
| **IC280887** | L53 | 33^MS^ | 14.2^MR^ | | 0 ^HR^ | 20 ^MR^ | 100 ^S^ | 20 ^MR^ | 0 ^HR^ |
| **IC201553** | L54 | 85^S^ | 50^MS^ | | 28.5 ^MS^ | 20 ^MR^ | 25 ^MS^ | 12.5 ^MR^ | 87 ^S^ |
| **EC303712** | L55 | 85^S^ | 62.5^S^ | | 0 ^HR^ | 14.2 ^MR^ | 0 ^HR^ | 0 ^HR^ | 85 ^S^ |
| **EC299645** | L56 | 100^S^ | 20^MR^ | | 12.5^MR^ | 14.2 ^MR^ | 0 ^HR^ | 20 ^MR^ | 100 ^S^ |
| **IC201537** | L57 | 37^MS^ | 42^MS^ | | 0 ^HR^ | 0 ^HR^ | 0 ^HR^ | 0 ^HR^ | 100 ^S^ |
| **IC384447** | L58 | 12.5^MR^ | 100^S^ | | 0 ^HR^ | 66.6 ^S^ | 100 ^S^ | - | 87 ^S^ |
| **IC53238** | L59 | 42^MS^ | 62.5^S^ | | 0 ^HR^ | 12.5 ^MR^ | 14 ^MR^ | - | 87 ^S^ |
| **IC424864** | L60 | 50^MS^ | 25^MS^ | | 14.2 ^MR^ | 25 ^MS^ | 75 ^S^ | 0 ^HR^ | 100^S^ |
| **IC346268** | L61 | 25^MS^ | 12.5^MR^ | | 16.6 ^MR^ | 25 ^MS^ | 25 ^MS^ | 0 ^HR^ | 0 ^HR^ |
| **IC616579** | L62 | 0^HR^ | 0^HR^ | | 50 ^MS^ | 40 ^MS^ | 33.3 ^MS^ | 14 ^MR^ | 25 ^MS^ |
| **IC201694** | L63 | 0^HR^ | 25^MS^ | | 20 ^MR^ | 28.5 ^MS^ | 50 ^MS^ | 0 ^HR^ | 16 ^MR^ |
| **IC241447** | L64 | 87^S^ | 50^MS^ | | 60 ^S^ | 57.1 ^S^ | 40 ^MS^ | 0 ^HR^ | 85 ^S^ |
| **IC201561** | L65 | 40^MS^ | 0^HR^ | | 28.5 ^MS^ | 14.2 ^MR^ | 0 ^HR^ | 0 ^HR^ | 57 ^S^ |
| **NC62506** | L66 | 28^MS^ | 100^S^ | | 14 ^MR^ | 28.5 ^MS^ | 14 ^MR^ | 25 ^MS^ | 77 ^S^ |
| **IC241532** | L67 | 85^S^ | 62.7^S^ | | 66.6 ^S^ | 83.3 ^S^ | 37.5 ^MS^ | 100 ^S^ | 85 ^S^ |
| **EC267595A** | L68 | 28^MS^ | 25^MS^ | | 40 ^MS^ | 50 ^MS^ | 12.5 ^MR^ | 0 ^HR^ | 0 ^HR^ |
| **IC717520** | L69 | 33^MS^ | 100^S^ | | 100 ^S^ | 83.3 ^S^ | 40 ^MS^ | 25 ^MS^ | 75 ^S^ |
| **EC78452** | L70 | 100^S^ | 33^MS^ | | 0 ^HR^ | 12.2 ^MR^ | 100 ^S^ | 14 ^MR^ | 100 ^S^ |
| **EC718473** | L71 | 50^MS^ | 0^HR^ | | 0 ^HR^ | 0 ^HR^ | 50 ^MS^ | 0 ^HR^ | 0 ^HR^ |
| **EC718659** | L72 | 0^HR^ | 100^S^ | | 0 ^HR^ | 100 ^S^ | 0 ^HR^ | 100 ^S^ | 0 ^HR^ |
| **EC718617** | L73 | 40^MS^ | 50^MS^ | | 0 ^HR^ | 25 ^MS^ | 25 ^MS^ | 0 ^HR^ | 0 ^HR^ |
| **EC718620** | L74 | 33^MS^ | 0^HR^ | | 0 ^HR^ | 0 ^HR^ | 0 ^HR^ | 33.3 ^MS^ | 0 ^HR^ |
| **EC718622** | L75 | 0^HR^ | 66^S^ | | 25 ^MS^ | 75 ^S^ | 100 ^S^ | 50 ^MS^ | 0 ^HR^ |
| **EC718234** | L76 | - | 0^HR^ | | 0 ^HR^ | 0 ^HR^ | - | - | 0 ^HR^ |
| **EC718516** | L77 | 0^HR^ | 100^S^ | | 100 ^S^ | 20 ^MR^ | 25 ^MS^ | 100^S^ | 50 ^MS^ |
| **EC718623** | L78 | 50^MS^ | 100^S^ | | 0 ^HR^ | 25 ^MS^ | 33.3 ^MS^ | 0 ^HR^ | 80 ^S^ |
| **EC718615** | L79 | 87.5^S^ | 100^S^ | | 25^MS^ | 100 ^S^ | 100 ^S^ | 50 ^MS^ | 0 ^HR^ |
| **EC718287** | L80 | 100^S^ | 33.3^MS^ | | 0 ^HR^ | 50 ^MS^ | 40 ^MS^ | 87.5 ^S^ | 100 ^S^ |
| **EC718694** | L81 | 50^MS^ | 0^HR^ | | 33.3 ^MS^ | 0 ^HR^ | 0 ^HR^ | 66.6 ^S^ | 0^HR^ |
| **EC718312** | L82 | 87.5^S^ | 50^MS^ | | 25 ^MS^ | 100 ^S^ | 25 ^MS^ | 33.3 ^MS^ | 50 ^MS^ |
| **EC714243** | L83 | 0^HR^ | 0^HR^ | | 0 ^HR^ | 0 ^HR^ | 0 ^HR^ | 0 ^HR^ | 0 ^HR^ |
| **EC718311** | L84 | 0^HR^ | 50^MS^ | | 80 ^S^ | 100 ^S^ | 75 ^S^ | 50 ^MS^ | 0 ^HR^ |
| **EC718252** | L85 | 100^S^ | 100^S^ | | 0 ^HR^ | 75 ^S^ | 100 ^S^ | 100 ^S^ | 100 ^S^ |
| **EC718690** | L86 | 0^HR^ | 0^HR^ | | 0 ^HR^ | 0 ^HR^ | 33.3 ^MS^ | 0 ^HR^ | 40 ^MS^ |
| **EC718692** | L87 | 66^S^ | 0^HR^ | | 0 ^HR^ | 33.3 ^MS^ | 33.3 ^MS^ | 0 ^HR^ | 50 ^MS^ |
| **EC718250** | L88 | 0^HR^ | 50^MS^ | | 0 ^HR^ | 0 ^HR^ | 50 ^MS^ | 0 ^HR^ | 0 ^HR^ |
| **EC718242** | L89 | 0^HR^ | 0^HR^ | | 33.3 ^MS^ | 50 ^MS^ | 33.3 ^MS^ | 33.3 ^MS^ | 25 ^MS^ |
| **EC718238** | L90 | 33^MS^ | 0^HR^ | | 0 ^HR^ | 0 ^HR^ | 0 ^HR^ | 0 ^HR^ | 0 ^HR^ |
| **EC718241** | L91 | 0^HR^ | 100^S^ | | 33.3 ^MS^ | 0 ^HR^ | 50 ^MS^ | 0 ^HR^ | 0 ^HR^ |
| **EC718253** | L92 | 100^S^ | 0^HR^ | | 50^MS^ | 87.5 ^S^ | 100 ^S^ | 75 ^S^ | 100 ^S^ |
| **EC664961** | L93 | 0^HR^ | 0 ^HR^ | | 0 ^HR^ | 100 ^S^ | 0 ^HR^ | 0 ^HR^ | 0 ^HR^ |
| **EC718326** | L94 | 50^MS^ | 0 ^HR^ | | 0 ^HR^ | 87.5 ^S^ | 0 ^HR^ | 0 ^HR^ | 0 ^HR^ |
| **EC718331** | L95 | 0^HR^ | 0 ^HR^ | | 0 ^HR^ | 50^MS^ | 0 ^HR^ | 0 ^HR^ | 0 ^HR^ |
| **EC718329** | L96 | 0^HR^ | 50^MS^ | | 50^MS^ | 0 ^HR^ | 0 ^HR^ | 0 ^HR^ | 25 |
| **EC718330** | L97 | 0^HR^ | 0 ^HR^ | | 0 ^HR^ | - | 0 ^HR^ | - | 0 ^HR^ |
| **EC718315** | L98 | 0^HR^ | 33^MS^ | | 0 ^HR^ | 0 ^HR^ | 50^MS^ | 0 ^HR^ | 25^MS^ |
| **EC718335** | L99 | 0^HR^ | 0 ^HR^ | | 33.3^MS^ | 0 ^HR^ | 50^MS^ | 0 ^HR^ | 0 ^HR^ |
| **EC718314** | L100 | 100^S^ | 87.5^S^ | | 50^MS^ | 0^HR^ | 66.6 ^S^ | 50^MS^ | 100 ^S^ |
| **PL639** | R check | 25^MS^ | 25^MS^ | | 12^MR^ | 14^MR^ | 12^MR^ | 14^MR^ | 14^MR^ |
| **L-9-12** | S check | 100^S^ | 50^S^ | | 50^S^ | 66^S^ | 75^S^ | 66^S^ | 75^S^ |

L1-L70- *L. c. subsp. culinaris;* L71-72- *L. c. subsp. tomentosus;* L73-79- *L. c subsp. orientalis;* L80- L84- *L. c. subsp. odemensis;* L85- L87- *L. lamottei;* L88-L93- *L. nigricans;* L94-L100- *L. ervoides.* HR: Highly resistant; R: resistant; MR: Moderately resistant; MS: Moderately susceptible; S: Susceptible. Dash represents accession were not screened for respective race.

**Supplementary Table 4.** Accession number obtained for isolated lentil RGA and result of similarity search between LRGA and known R gene from other plant species using BLASTX.

| **Lentil RGA** | **Accession number** | **Similarity with other R gene** | **Maximum identity** | **E-value** |
| --- | --- | --- | --- | --- |
| **LcRGA1** | ON367522 | RUN1 disease resistance protein *Mediacago truncatula* | 83 | 7e-125 |
| **LcRGA2** | ON420341 | RUN1 disease resistance protein *M. truncatula* | 99 | 5e-186 |
| **LcRGA3** | ON367523 | RUN1 disease resistance protein *M. truncatula* | 83 | 2e-124 |
| **LcRGA4** | ON420342 | RUN1 disease resistance protein *M. truncatula* | 86 | 5e-146 |
| **LcRGA5** | ON420343 | RUN1 disease resistance protein *M. truncatula* | 88 | 2e-164 |
| **LcRGA6** | ON367524 | RUN1 disease resistance protein *M. truncatula* | 83 | 2e-124 |
| **LcRGA7** | ON367525 | RUN1 disease resistance protein *M. truncatula* | 83 | 2e-124 |
| **LcRGA8** | ON367526 | RUN1 disease resistance protein *M. truncatula* | 83 | 2e-124 |
| **LcRGA9** | ON367527 | RUN1 disease resistance protein *M. truncatula* | 83 | 2e-124 |
| **LcRGA10** | ON381744 | N disease resistance protein of *Trifolium partense* | 78 | 3e-79 |
| **LcRGA11** | ON381743 | RUN1 disease resistance protein *M. truncatula* | 76 | 3e-64 |
| **LcRGA12** | ON367528 | RUN1 disease resistance protein *M. truncatula* | 83 | 5e-126 |
| **LcRGA13** | ON367529 | RUN1 disease resistance protein *M. truncatula* | 83 | 1e-122 |
| **LcRGA14** | ON420344 | N disease resistance protein of *T. partense* | 88 | 5e-166 |
| **LcRGA15** | ON367530 | RUN1 disease resistance protein *M. truncatula* | 82 | 2e-119 |
| **LoRGA1** | ON381745 | RUN1 disease resistance protein *M. truncatula* | 90 | 0 |
| **LoRGA2** | ON399215 | RUN1 disease resistance protein *M. truncatula* | 90 | 0 |
| **LoRGA3** | ON399211 | RUN1 disease resistance protein *M. truncatula* | 91 | 0 |
| **LoRGA4** | ON399212 | RUN1 disease resistance protein *M. truncatula* | 90 | 0 |
| **LoRGA5** | ON399213 | RUN1 disease resistance protein *M. truncatula* | 90 | 0 |
| **LoRGA6** | ON399214 | RUN1 disease resistance protein *M. truncatula* | 90 | 0 |
| **LoRGA7** | ON409891 | RUN1 disease resistance protein *M. truncatula* | 90 | 6e-167 |
| **LoRGA8** | ON409892 | RUN1 disease resistance protein *M. truncatula* | 90 | 2e-167 |
| **LoRGA9** | ON409893 | RUN1 disease resistance protein *M. truncatula* | 90 | 0 |
| **LoRGA10** | ON409894 | RUN1 disease resistance protein *M. truncatula* | 90 | 2e-167 |
| **LoRGA11** | ON409895 | RUN1 disease resistance protein *M. truncatula* | 90 | 2e-167 |
| **LoRGA12** | ON409896 | RUN1 disease resistance protein *M. truncatula* | 90 | 0 |
| **LoRGA13** | ON409897 | RUN1 disease resistance protein *M. truncatula* | 90 | 0 |
| **LoRGA14** | ON409898 | RUN1 disease resistance protein *M. truncatula* | 90 | 0 |
| **LoRGA15** | ON409899 | RUN1 disease resistance protein *M. truncatula* | 89 | 3e-160 |
| **LnRGA1** | ON420345 | N disease resistance protein of *T. partense* | 89 | 2e-169 |
| **LnRGA2** | ON420346 | RPP13 disease resistance protein *M. truncatula* | 81 | 2e-113 |
| **LnRGA3** | ON420347 | RUN1 disease resistance protein *M. truncatula* | 90 | 0 |
| **LnRGA4** | ON420348 | RUN1 disease resistance protein *M. truncatula* | 90 | 2e-167 |
| **LnRGA5** | ON420349 | RUN1 disease resistance protein *M. truncatula* | 88 | 5e-166 |
| **LnRGA6** | ON454553 | RUN1 disease resistance protein *M. truncatula* | 90 | 5e-165 |
| **LnRGA7** | ON454554 | N disease resistance protein of *T. partense* | 88 | 5e-166 |
| **LnRGA8** | ON454555 | RUN1 disease resistance protein *M. truncatula* | 90 | 5e-165 |
| **LnRGA9** | ON454556 | RPP13 disease resistance protein *M. truncatula* | 81 | 2e-109 |
| **LnRGA10** | ON454557 | RUN1 disease resistance protein *M. truncatula* | 80 | 3e-105 |
| **LnRGA11** | ON454558 | RUN1 disease resistance protein *M. truncatula* | 90 | 0 |
| **LnRGA12** | ON454559 | RUN1 disease resistance protein *M. truncatula* | 90 | 2e-166 |
| **LnRGA13** | ON454562 | RUN1 disease resistance protein *M. truncatula* | 88 | 1e-166 |
| **LnRGA14** | ON454560 | RUN1 disease resistance protein *M. truncatula* | 90 | 2e-167 |
| **LnRGA15** | ON454561 | N disease resistance protein of *T. partense* | 89 | 2e-169 |

| **Lentil RGA**  **Supplementary Table 5**. Prediction of structural analog, molecular, biological and cellular function of isolated Lentil RGA using I-TASSER | **Structural analogue in PDB protein** | **Function** | **Molecular function** | **Biological function** | **Cellular function** |
| --- | --- | --- | --- | --- | --- |
| **LcRGA1** | Plant NLR RPP1 tetramer in complex with ATR1 | AAA ATPases | ATP BINDING | DNA replication | Cellular component |
| **LcRGA2** | Activated Roq1 resistosome directly recognizing the pathogen effector XopQ in tobacco | AAA ATPases | ATP BINDING | DNA replication | DNA polymerase III complex |
| **LcRGA3** | Plant NLR resistosome conferring immunity | AAA ATPases | ATP BINDING | DNA replication | Cellular component |
| **LcRGA4** | Plant NLR RPP1 tetramer in complex with ATR1 | AAA ATPases | ATP BINDING | DNA replication | DNA polymerase III complex |
| **LcRGA5** | Plant NLR RPP1 tetramer in complex with ATR1 | AAA ATPases | ATP BINDING | DNA replication | Cellular component |
| **LcRGA6** | Plant NLR RPP1 tetramer in complex with ATR1 | AAA ATPases | ATP BINDING | DNA replication | Cellular component |
| **LcRGA7** | Plant NLR RPP1 tetramer in complex with ATR1 | AAA ATPases | ATP BINDING | DNA replication | Cellular component |
| **LcRGA8** | Plant NLR RPP1 tetramer in complex with ATR1 | AAA ATPases | ATP BINDING | DNA replication | Cellular component |
| **LcRGA9** | Plant NLR RPP1 tetramer in complex with ATR1 | AAA ATPases | ATP BINDING | DNA replication | Cellular component |
| **LcRGA10** | Plant NLR RPP1 tetramer in complex with ATR1 | AAA ATPases | ATP BINDING | DNA replication | Cellular component |
| **LcRGA11** | Activated Roq1 resistosome directly recognizing the pathogen effector XopQ | AAA ATPases | ATP BINDING | DNA replication | Cellular component |
| **LcRGA12** | Plant NLR RPP1 tetramer in complex with ATR1 | AAA ATPases | ATP BINDING | DNA replication | Cellular component |
| **LcRGA13** | Plant NLR RPP1 tetramer in complex with ATR1 | AAA ATPases | ATP BINDING | DNA replication | Cellular component |
| **LcRGA14** | Plant NLR RPP1 tetramer in complex with ATR1 | AAA ATPases | ATP BINDING | DNA replication | Cellular component |
| **LcRGA15** | Plant NLR PP1 tetramer in complex with ATR1 | AAA ATPases | ATP BINDING | DNA replication | DNA polymerase III complex |
| **LoRGA1** | Plant NLR RPP1 tetramer in complex with ATR1 | AAA ATPases | ATP BINDING | DNA replication | DNA polymerase III complex |
| **LoRGA2** | Activated Roq1 resistosome directly recognizing the pathogen effector XopQ | AAA ATPases | ATP BINDING | DNA replication | DNA polymerase III complex |
| **LoRGA3** | Activated Roq1 resistosome directly recognizing the pathogen effector XopQ | AAA ATPases | ATP BINDING | DNA replication | DNA polymerase III complex |
| **LoRGA4** | Activated Roq1 resistosome directly recognizing the pathogen effector XopQ | AAA ATPases | ATP BINDING | DNA replication | Cellular component |
| **LoRGA5** | Activated Roq1 resistosome directly recognizing the pathogen effector XopQ | AAA ATPases | ATP BINDING | DNA replication | DNA polymerase III complex |
| **LoRGA6** | Activated Roq1 resistosome directly recognizing the pathogen effector XopQ | AAA ATPases | ATP BINDING | DNA replication | DNA polymerase III complex |
| **LoRGA7** | Activated Roq1 resistosome directly recognizing the pathogen effector XopQ | AAA ATPases | ATP BINDING | intrinsic apoptotic signaling pathway | Golgi complex |
| **LoRGA8** | Plant NLR RPP1 tetramer in complex with ATR1 | AAA ATPases | ATP BINDING | DNA replication | DNA polymerase III complex |
| **LoRGA9** | Activated Roq1 resistosome directly recognizing the pathogen effector XopQ | AAA ATPases | ATP BINDING | DNA replication | Soluble fraction |
| **LoRGA10** | Activated Roq1 resistosome directly recognizing the pathogen effector XopQ | AAA ATPases | ATP BINDING | DNA replication | DNA polymerase III complex |
| **LoRGA11** | Activated Roq1 resistosome directly recognizing the pathogen effector XopQ | AAA ATPases | ATP BINDING | DNA replication | DNA polymerase III complex |
| **LoRGA12** | Activated Roq1 resistosome directly recognizing the pathogen effector XopQ | AAA ATPases | ATP BINDING | DNA replication | DNA polymerase III complex |
| **LoRGA13** | Activated Roq1 resistosome directly recognizing the pathogen effector XopQ | AAA ATPases | ATP BINDING | DNA replication | DNA polymerase III complex |
| **LoRGA14** | Activated Roq1 resistosome directly recognizing the pathogen effector XopQ | AAA ATPases | ATP BINDING | DNA replication | DNA polymerase III complex |
| **LoRGA15** | Activated Roq1 resistosome directly recognizing the pathogen effector XopQ | AAA ATPases | ATP BINDING | DNA replication | mitochondria |
| **LnRGA1** | Plant NLR resistosome membrane protein/ structure of plant NLR RPP1 tetramer in complex with ATR1 | AAA ATPases | ATP BINDING | DNA replication | DNA polymerase III complex |
| **LnRGA2** | Plant NLR resistosome in complex with ATR1 | AAA ATPases | ATP BINDING | DNA replication | DNA polymerase III complex |
| **LnRGA3** | Activated Roq1 resistosome directly recognizing the pathogen effector XopQ | AAA ATPases | ATP BINDING | DNA replication | cystol |
| **LnRGA4** | Activated Roq1 resistosome directly recognizing the pathogen effector XopQ | AAA ATPases | ATP BINDING | DNA replication | cystol |
| **LnRGA5** | Activated Roq1 resistosome directly recognizing the pathogen effector XopQ | AAA ATPases | ATP BINDING | DNA replication | DNA polymerase III complex |
| **LnRGA6** | Activated Roq1 resistosome directly recognizing the pathogen effector XopQ | AAA ATPases | ATP BINDING | DNA replication | cystol |
| **LnRGA7** | Plant NLR RPP1 tetramer in complex with ATR1 | AAA ATPases | ATP BINDING | DNA replication | DNA polymerase III complex |
| **LnRGA8** | Activated Roq1 resistosome directly recognizing the pathogen effector XopQ | AAA ATPases | ATP BINDING | DNA replication | DNA polymerase III complex |
| **LnRGA9** | Plant NLR RPP1 tetramer in complex with ATR1 | AAA ATPases | ATP BINDING | DNA replication | Perinuclear region of cytoplasm |
| **LnRGA10** | Plant NLR RPP1 tetramer in complex with ATR1 | AAA ATPases | ATP BINDING | DNA replication | Cellular component |
| **LnRGA11** | Activated Roq1 resistosome directly recognizing the pathogen effector XopQ | AAA ATPases | ATP BINDING | DNA replication | mitochondria |
| **LnRGA12** | Activated Roq1 resistome directly recognizing the pathogen effector XopQ | AAA ATPases | ATP BINDING | DNA replication | DNA polymerase III complex |
| **LnRGA13** | Plant NLR RPP1 tetramer in complex with ATR1 | AAA ATPases | ATP BINDING | DNA replication | DNA polymerase III complex |
| **LnRGA14** | Activated Roq1 resistosome directly recognizing the pathogen effector XopQ | AAA ATPases | ATP BINDING | DNA replication | Cellular component |
| **LnRGA15** | Activated Roq1 resistosome directly recognizing the pathogen effector XopQ | AAA ATPases | ATP BINDING | DNA replication | DNA polymerase III complex |

**Supplementary Table 6**. Predicted of Active binding site of RGA and its corresponding amino acid, its solubility and conserved motif

| **Lentil RGA** | **Active binding site (ABS)** | **Amino acid corresponding ABS** | **Predicted solvent accessibility of ABS** | **Motif corresponding to ABS** |
| --- | --- | --- | --- | --- |
| **LcRGA1** | 4, 6, 110 | G, T, S | 3, 2, 1 | P-loop, Kinase3 |
| **LcRGA2** | 109, 111 | S, N | 1, 1 | Kinase3 |
| **LcRGA3** | 4, 6, 83, 110 | G, T, N, S | 3, 2, 1, 1 | P-loop, Kinase 2, Kinase3 |
| **LcRGA4** | - | - | - |  |
| **LcRGA5** | - | - | - |  |
| **LcRGA6** | 4, 6, 82 | G, T, D | 3, 2, 0 | P-loop, Kinase2 |
| **LcRGA7** | 4, 6, 82 | G, T, D | 3, 2, 0 | P-loop, Kinase2 |
| **LcRGA8** | 4, 6, 27, 83 | G, T, D, N | 3, 2, 3, 1 | P-loop, TIR-RNBS, Kinase2 |
| **LcRGA9** | 4, 6, 83, 110 | G, T, N, S | 3, 2, 1, 1 | P-loop, Kinase 2, Kinase3 |
| **LcRGA10** | 109 | S | 1 | Kinase3 |
| **LcRGA11** | - | - | - | - |
| **LcRGA12** | 4, 6, 27, 83 | G, T, D, N | 3, 2, 3, 1 | P-loop, TIR-RNBS, Kinase2 |
| **LcRGA13** | 4, 6, 82 | G, T, D | 3, 2, 0 | P-loop, Kinase2 |
| **LcRGA14** | 4, 6 | G, T | 3, 2 | P-loop |
| **LcRGA15** | 4, 6, 27, 83 | G, T, D, N | 3, 2, 3, 1 | P-loop, TIR-RNBS, Kinase2 |
| **LoRGA1** | 4, 6, 27, 82 | G, T, D, N | 3, 2, 3, 1 | P-loop, TIR-RNBS, Kinase2 |
| **LoRGA2** | 4, 6, 27 | G, T, D | 3, 2, 3 | P-loop, Kinase2 |
| **LoRGA3** | 4, 6, 81, 135 | G, T, D, L | 3, 2, 0, 1 | P-loop, Kinase2, RNBS-C |
| **LoRGA4** | 4, 6, 27, 82 | G, T, D, N | 3, 2, 3, 1 | P-loop, TIR-RNBS, Kinase2 |
| **LoRGA5** | 4, 6, 27, 82 | G, T, D, N | 3, 2, 3, 1 | P-loop, TIR-RNBS, Kinase2 |
| **LoRGA6** | 4, 6, 27, 82 | G, T, D, N | 3, 2, 3, 1 | P-loop, TIR-RNBS, Kinase2 |
| **LoRGA7** | 3 | G | 4 | P-loop |
| **LoRGA8** | 4, 6, 27 | G, T, D | 3, 2, 3 | P-loop, TIR-RNBS |
| **LoRGA9** | 4, 6, 27, 82 | G, T, D, N | 3, 2, 3, 1 | P-loop, TIR-RNBS, Kinase2 |
| **LoRGA10** | 4, 6, 27, 82 | G, T, D, N | 3, 2, 3, 1 | P-loop, TIR-RNBS, Kinase2 |
| **LoRGA11** | 4, 6, 27, 82 | G, T, D, N | 3, 2, 3, 1 | P-loop, TIR-RNBS, Kinase2 |
| **LoRGA12** | - | - | - | - |
| **LoRGA13** | 4, 6, 27, 82 | G, T, D, N | 3, 2, 3, 1 | P-loop, TIR-RNBS, Kinase2 |
| **LoRGA14** | 4, 6, 27, 82 | G, T, D, N | 3, 2, 3, 1 | P-loop, TIR-RNBS, Kinase2 |
| **LoRGA15** | 4, 6, 27, 82 | G, T, D, N | 3, 2, 3, 1 | P-loop, TIR-RNBS, Kinase2 |
| **LnRGA1** | 114 | L | 0 | Kinase3 |
| **LnRGA2** | 4, 6, 78, 108 | G, T, D, T |  | P-loop, Kinase2, Kinase3 |
| **LnRGA3** | 4, 6, 27, 82 | G, T, D, N | 3, 2, 3, 1 | P-loop, TIR-RNBS, Kinase2 |
| **LnRGA4** | 4, 6, 27 | G, T, D | 3, 2, 3 | P-loop, TIR-RNBS |
| **LnRGA5** | - | - | - | - |
| **LnRGA6** | 4, 6, 27, 82 | G, T, D, N | 3, 2, 3, 1 | P-loop, TIR-RNBS, Kinase2 |
| **LnRGA7** | - | - | - | - |
| **LnRGA8** | 4, 6, 81, 135 | G, T, D, L | 3, 2, 0, 1 | P-loop, Kinase2, RNBS-C |
| **LnRGA9** | 4, 6, 78, 167 | G, T, D, G | 4, 2, 0, 4 | P-loop, Kinase2, GLPL |
| **LnRGA10** | 4, 6, 27, 83 | G, T, D, N | 3, 2, 3, 1 | P-loop, TIR-RNBS, Kinase2 |
| **LnRGA11** | 4, 6, 27, 82 | G, T, D, N | 3, 2, 3, 1 | P-loop, TIR-RNBS, Kinase2 |
| **LnRGA12** | 4, 6, 27, 82 | G, T, D, N | 3, 2, 3, 1 | P-loop, TIR-RNBS, Kinase2 |
| **LnRGA13** | 4, 6, 78, 167 | G, T, D, G |  | P-loop, Kinase2, GLPL |
| **LnRGA14** | 4, 6, 81, 135 | G, T, D, L | 3, 2, 0, 1 | P-loop, Kinase2, RNBS-C |
| **LnRGA15** | - | - | - | - |
| ‘-’Dash represents no Active binding sites were predicted | | | | |

**Supplementary Table 7**. Predicted C-Score, TM-Score and RMSD of RGA tertiary structure

| **Lentil RGA** | **C-Score** | **TM-Score** | **RMSD** |
| --- | --- | --- | --- |
| **LcRGA1** | 0.65 | 0.80±0.09 | 3.7±2.5Å |
| **LcRGA2** | 0.43 | 0.77±0.10 | 4.1±2.8Å |
| **LcRGA3** | 0.58 | 0.79±0.09 | 3.9±2.6Å |
| **LcRGA4** | 0.61 | 0.80±0.09 | 3.8±2.6Å |
| **LcRGA5** | 0.35 | 0.76±0.10 | 4.3±2.9Å |
| **LcRGA6** | 0.50 | 0.78±0.10 | 4.0±2.7Å |
| **LcRGA7** | 0.50 | 0.78±0.10 | 4.0±2.7Å |
| **LcRGA8** | 0.50 | 0.78±0.10 | 4.0±2.7Å |
| **LcRGA9** | 0.58 | 0.79±0.09 | 3.9±2.6Å |
| **LcRGA10** | 0.36 | 0.76±0.10 | 4.3±2.9Å |
| **LcRGA11** | 0.33 | 0.76±0.10 | 4.3±2.9Å |
| **LcRGA12** | 0.47 | 0.78±0.10 | 4.1±2.7Å |
| **LcRGA13** | 0.51 | 0.78±0.10 | 4.0±2.7Å |
| **LcRGA14** | 0.31 | 0.75±0.10 | 4.4±2.9Å |
| **LcRGA15** | 0.51 | 0.78±0.10 | 4.0±2.7Å |
| **LoRGA1** | 0.51 | 0.78±0.10 | 4.0±2.7Å |
| **LoRGA2** | 0.31 | 0.75±0.10 | 4.4±2.9Å |
| **LoRGA3** | 0.35 | 0.76±0.10 | 4.3±2.9Å |
| **LoRGA4** | 0.46 | 0.77±0.10 | 4.1±2.7Å |
| **LoRGA5** | 0.44 | 0.77± 0.10 | 4.1 ±2.8 Å |
| **LoRGA6** | 0.42 | 0.77± 0.10 | 4.2 ±2.8 Å |
| **LoRGA7** | 0.42 | 0.77± 0.10 | 4.2 ±2.8 Å |
| **LoRGA8** | 0.64 | 0.80±0.09 | 3.7±2.6Å |
| **LoRGA9** | 0.46 | 0.77±0.10 | 4.1±2.7Å |
| **LoRGA10** | 0.59 | 0.79±0.09 | 3.8±2.6Å |
| **LoRGA11** | 0.41 | 0.77±0.10 | 4.2 ±2.8 Å |
| **LoRGA12** | 0.41 | 0.77±0.10 | 4.2±2.8Å |
| **LoRGA13** | 0.51 | 0.78±0.10 | 4.0±2.7Å |
| **LoRGA14** | 0.44 | 0.77± 0.10 | 4.1 ±2.8 Å |
| **LoRGA15** | 0.41 | 0.77±0.10 | 4.2±2.8Å |
| **LnRGA1** | 0.41 | 0.77±0.10 | 4.2±2.8Å |
| **LnRGA2** | 0.41 | 0.77±0.10 | 4.2±2.8Å |
| **LnRGA3** | 0.62 | 0.80 ±0.9 | 3.8 ±2.6 Å |
| **LnRGA4** | 0.42 | 0.77±0.10 | 4.2±2.8Å |
| **LnRGA5** | 0.33 | 0.76±0.10 | 4.3±2.9Å |
| **LnRGA6** | 0.41 | 0.77±0.10 | 4.2±2.8Å |
| **LnRGA7** | 0.36 | 0.76±0.10 | 4.3±2.8Å |
| **LnRGA8** | 0.35 | 0.76±0.10 | 4.3±2.9Å |
| **LnRGA9** | 0.68 | 0.81 ±0.09 | 3.7±2.5 Å |
| **LnRGA10** | 0.50 | 0.78±0.10 | 4.0±2.7Å |
| **LnRGA11** | 0.42 | 0.70 ±0.10 | 4.2 ±2.8 Å |
| **LnRGA12** | 0.44 | 0.77± 0.10 | 4.1 ±2.8 Å |
| **LnRGA13** | 0.69 | 0.81 ±0.09 | 3.7 ±2.5 Å |
| **LnRGA14** | 0.41 | 0.77±0.10 | 4.2±2.8Å |
| **LnRGA15** | 0.36 | 0.76±0.10 | 4.3±2.8 Å |

**Supplementary Table 8.** Identification of Lentil R gene based on similarity search and prediction of lentil R gene structural analogue.

| **Lentil RGA** | **Lentil R gene** | **Structural analogue in PDB protein** |
| --- | --- | --- |
| **LcRGA1** | Lcu.2RBY.7g024020.1 | Plant NLR RPP1 tetramer in complex with ATR1 |
| **LcRGA2** | Lcu.2RBY.5g000580.1 | Plant NLR RPP1 tetramer in complex with ATR1 |
| **LcRGA3** | Lcu.2RBY.5g020180.1 | Plant NLR resistosome conferring immunity |
| **LcRGA4** | Lcu.2RBY.6g029790.1 | Plant NLR RPP1 tetramer in complex with ATR1 |
| **LcRGA5** | Lcu.2RBY.6g037340.1 | Plant NLR RPP1 tetramer in complex with ATR1 |
| **LcRGA6** | Lcu.2RBY.6g029790.1 | Plant NLR RPP1 tetramer in complex with ATR1 |
| **LcRGA7** | Lcu.2RBY.6g029790.1 | Plant NLR RPP1 tetramer in complex with ATR1 |
| **LcRGA8** | Lcu.2RBY.6g048640.1 | Plant NLR resistosome conferring immunity |
| **LcRGA9** | Lcu.2RBY.6g016620.1 | Plant NLR resistosome conferring immunity |
| **LcRGA10** | Lcu.2RBY.6g029790.1 | Plant NLR RPP1 tetramer in complex with ATR1 |
| **LcRGA11** | Lcu.2RBY.6g029760.1 | Plant NLR RPP1 tetramer in complex with ATR1 |
| **LcRGA12** | Lcu.2RBY.2g033550.1 | Plant NLR RPP1 tetramer in complex with ATR1 |
| **LcRGA13** | Lcu.2RBY.6g029790.1 | Plant NLR RPP1 tetramer in complex with ATR1 |
| **LcRGA14** | Lcu.2RBY.2g031390.1 | Plant NLR RPP1 tetramer in complex with ATR1 |
| **LcRGA15** | Lcu.2RBY.6g029790.1 | Plant NLR RPP1 tetramer in complex with ATR1 |
| **LoRGA1** | Lcu.2RBY.5g000580.1 | Plant NLR RPP1 tetramer in complex with ATR1 |
| **LoRGA2** | Lcu.2RBY.5g000580.1 | Plant NLR RPP1 tetramer in complex with ATR1 |
| **LoRGA3** | Lcu.2RBY.5g000580.1 | Plant NLR RPP1 tetramer in complex with ATR1 |
| **LoRGA4** | Lcu.2RBY.5g000580.1 | Plant NLR RPP1 tetramer in complex with ATR1 |
| **LoRGA5** | Lcu.2RBY.5g000580.1 | Plant NLR RPP1 tetramer in complex with ATR1 |
| **LoRGA6** | Lcu.2RBY.5g000580.1 | Plant NLR RPP1 tetramer in complex with ATR1 |
| **LoRGA7** | Lcu.2RBY.5g000580.1 | Plant NLR RPP1 tetramer in complex with ATR1 |
| **LoRGA8** | Lcu.2RBY.5g000580.1 | Plant NLR RPP1 tetramer in complex with ATR1 |
| **LoRGA9** | Lcu.2RBY.5g000580.1 | Plant NLR RPP1 tetramer in complex with ATR1 |
| **LoRGA10** | Lcu.2RBY.5g000580.1 | Plant NLR RPP1 tetramer in complex with ATR1 |
| **LoRGA11** | Lcu.2RBY.5g000580.1 | Plant NLR RPP1 tetramer in complex with ATR1 |
| **LoRGA12** | Lcu.2RBY.5g000580.1 | Plant NLR RPP1 tetramer in complex with ATR1 |
| **LoRGA13** | Lcu.2RBY.5g000580.1 | Plant NLR RPP1 tetramer in complex with ATR1 |
| **LoRGA14** | Lcu.2RBY.5g000580.1 | Plant NLR RPP1 tetramer in complex with ATR1 |
| **LoRGA15** | Lcu.2RBY.5g000580.1 | Plant NLR RPP1 tetramer in complex with ATR1 |
| **LnRGA1** | Lcu.2RBY.2g053640.1 | Plant NLR RPP1 tetramer in complex with ATR1 |
| **LnRGA2** | Lcu.2RBY.3g011370.1  Lcu.2RBY.3g013800.1 | Plant NLR resistosome conferring immunity |
| **LnRGA3** | Lcu.2RBY.5g000580.1 | Plant NLR RPP1 tetramer in complex with ATR1 |
| **LnRGA4** | Lcu.2RBY.2g033550.1 | Plant NLR RPP1 tetramer in complex with ATR1 |
| **LnRGA5** | Lcu.2RBY.L012800.1 | Plant NLR RPP1 tetramer in complex with ATR1 |
| **LnRGA6** | Lcu.2RBY.6g029760.1 | Plant NLR RPP1 tetramer in complex with ATR1 |
| **LnRGA7** | Lcu.2RBY.L012800.1 | Plant NLR RPP1 tetramer in complex with ATR1 |
| **LnRGA8** | Lcu.2RBY.2g053630.1 | Plant NLR RPP1 tetramer in complex with ATR1 |
| **LnRGA9** | Lcu.2RBY.3g011520.1  Lcu.2RBY.3g011540.1 | Plant NLR RPP1 tetramer in complex with ATR1 |
| **LnRGA10** | Lcu.2RBY.6g029760.1 | Plant NLR RPP1 tetramer in complex with ATR1 |
| **LnRGA11** | Lcu.2RBY.6g029760.1 | Plant NLR RPP1 tetramer in complex with ATR1 |
| **LnRGA12** | Lcu.2RBY.6g029760.1 | Plant NLR RPP1 tetramer in complex with ATR1 |
| **LnRGA13** | Lcu.2RBY.3g011620.1/  Lcu.2RBY.3g011390.1 | Plant NLR resistosome conferring immunity |
| **LnRGA14** | Lcu.2RBY.6g029760.1 | Plant NLR RPP1 tetramer in complex with ATR1 |
| **LnRGA15** | Lcu.2RBY.1g066550.1 | Plant NLR RPP1 tetramer in complex with ATR1 |


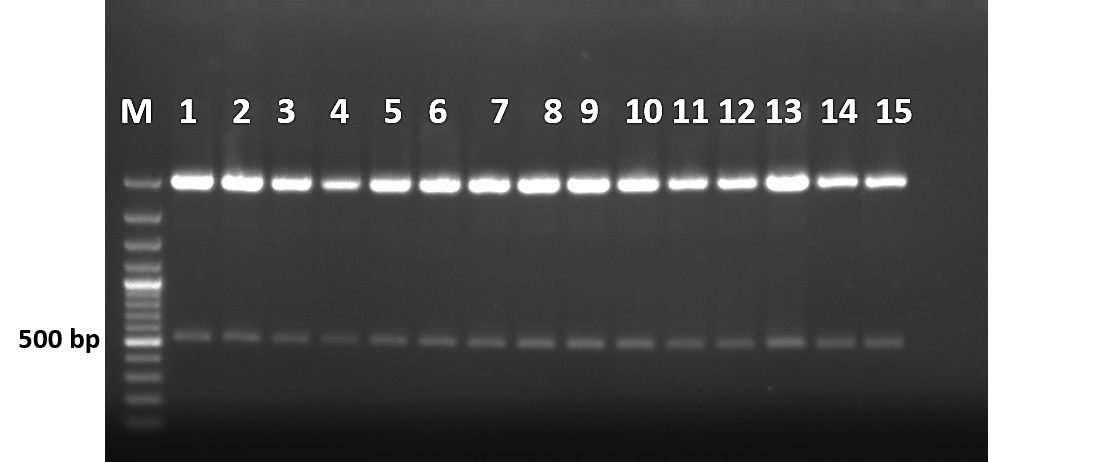


**Supplementary figure 1**. Restriction digestion profile of plasmid having resistance gene analogue as insert (͌500 bp) isolated from *Lens culinaris* sub sp. *culinaris* (L65) digested with EcoR1 enzyme ran on 1.2% agarose gel. M= 1kb ladder, lanes 1 to 15 consist of RGA, LcRGA1 to LcRGA15.


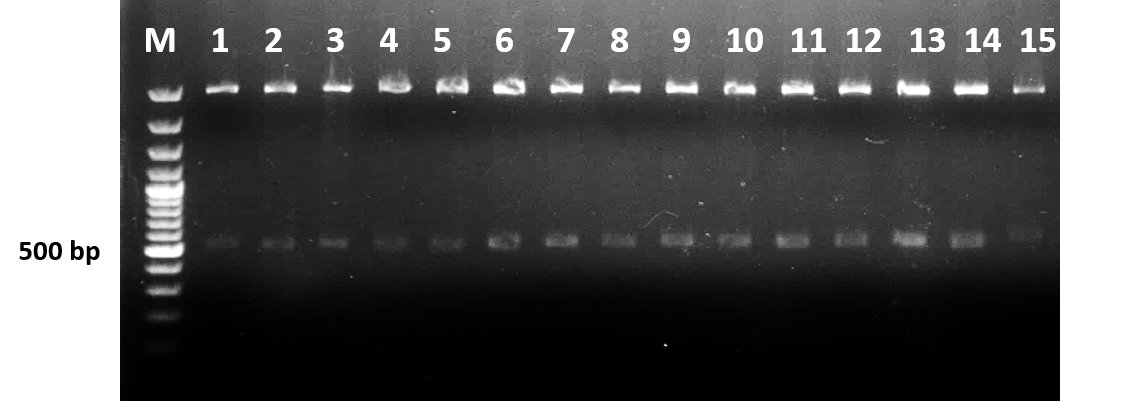


**Supplementary figure 2**. Restriction digestion profile of plasmid having resistance gene analogue as insert (͌500 bp) isolated from *Lens culinaris* sub sp. *odemensis* (L83) digested with EcoR1 enzyme ran on 1.2% agarose gel. Lane Mi is1kb ladder and lanes 1 to 15 consist of RGA, LoRGA1 to LoRGA15.


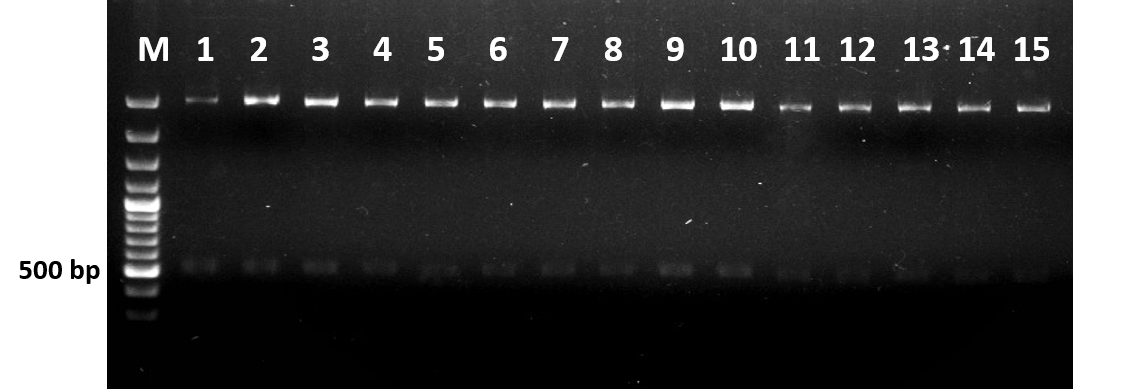


**Supplementary figure 3**. Restriction digestion profile of plasmid having resistance gene analogue as insert (͌500 bp) isolated from *Lens nigricans* (L90) digested with EcoR1 enzyme ran on 1.2% agarose gel. Lane M is 1kb ladder and lanes 1 to 15 consist of RGA, LnRGA1 to LnRGA15.

LcRGA1

LcRGA2

LcRGA3

LcRGA4

LcRGA5


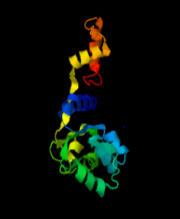

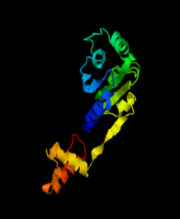

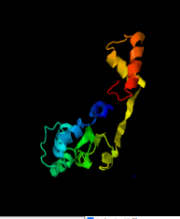


LcRGA6

LcRGA7

LcRGA8

LcRGA9

LcRGA10


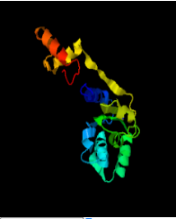

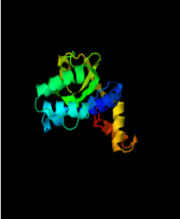

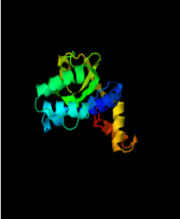

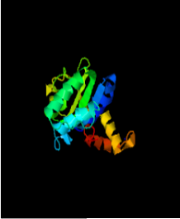

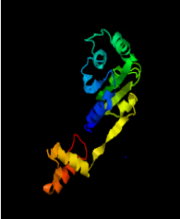

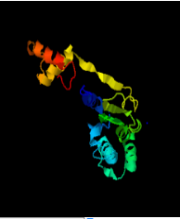

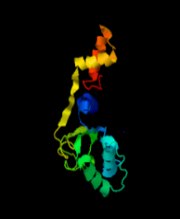

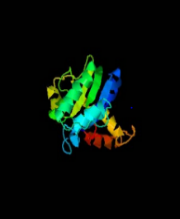


LoRGA5


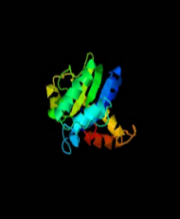

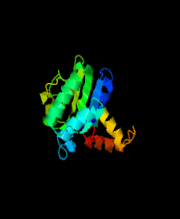

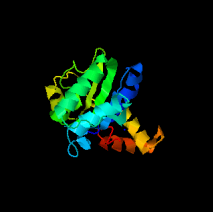

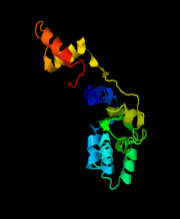

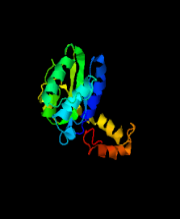


LoRGA6

LoRGA7

LoRGA8

LoRGA9

LoRGA10


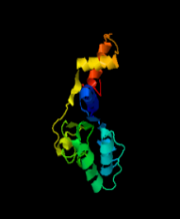

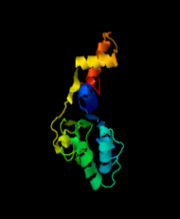


LoRGA11

LoRGA12


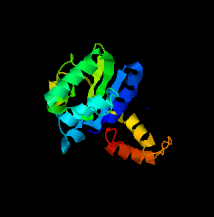

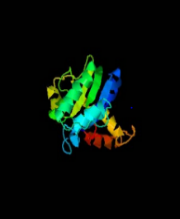

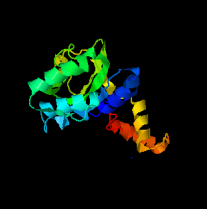


LoRGA13

LoRGA14

LoRGA15


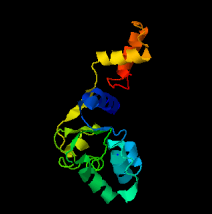


LnRGA1

LnRGA4


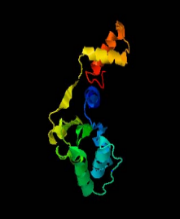

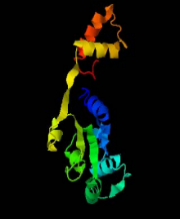

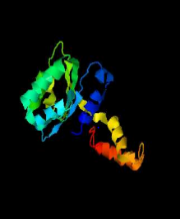


LnRGA2

LnRGA3


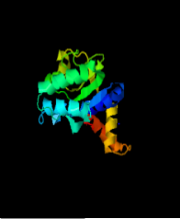

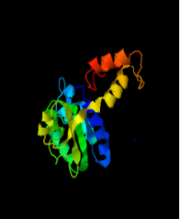

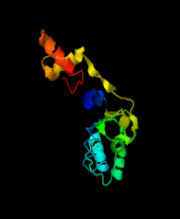

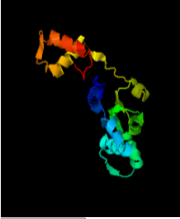


LcRGA11

LcRGA12

LcRGA13

LcRGA14

LcRGA15


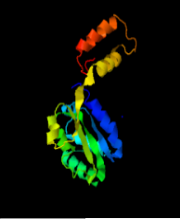

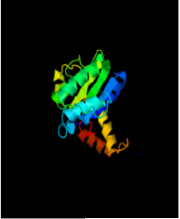


LoRGA1

LoRGA2

LoRGA3

LoRGA4


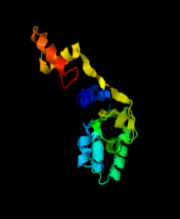

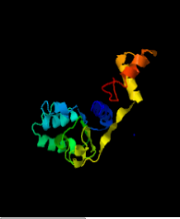

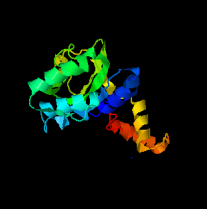

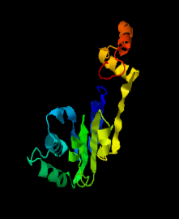


LnRGA5


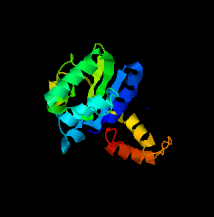

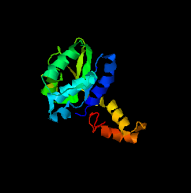

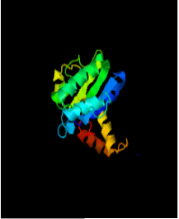

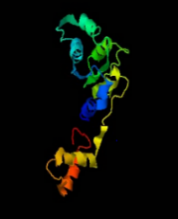

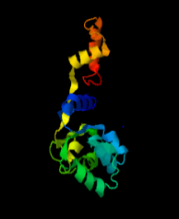


LnRGA6

LnRGA7

LnRGA8

LnRGA9

LnRGA10


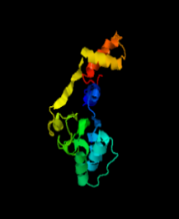

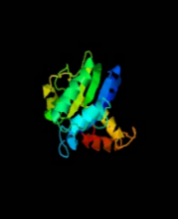

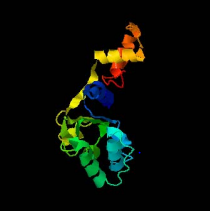

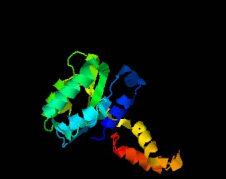


LnRGA11

LnRGA12

LnRGA13

LnRGA14

LnRGA15


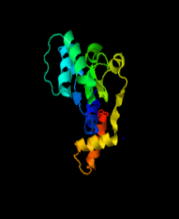


**Supplementary figure 4.** Tertiary structure of RGA predicted using online I-TASSER software. RGA, LcRGA1-15 isolated from Lens culinaris subsp. culinaris, LoRGA1-15 isolated from L. culinaris subsp. odemensis and LnRGA1-15 isolated from L. nigricans.


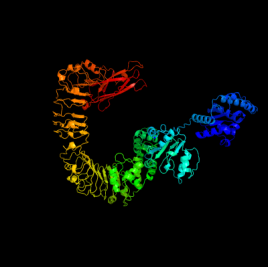


Lcu_2RBY_6g029760_1


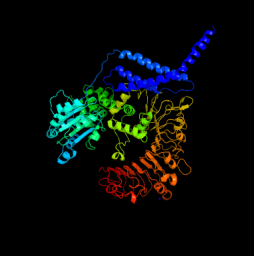


Lcu_2RBY_3g011620_1


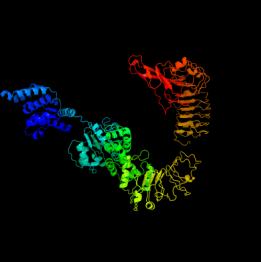


Lcu_2RBY_L012800_1


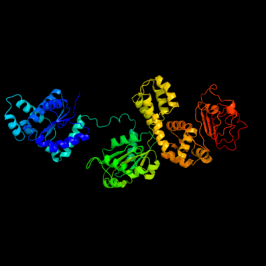


Lcu.2RBY.2g033550.1


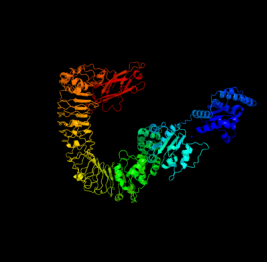


Lcu_2RBY_6g048640_1


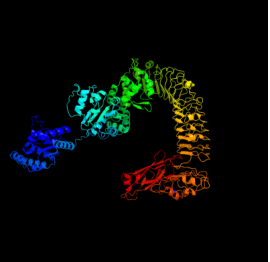


Lcu_2RBY_6g037340_1


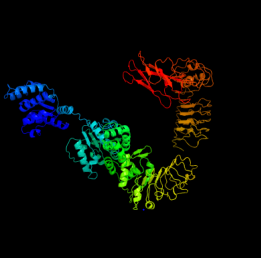


Lcu_2RBY_6g016620_1


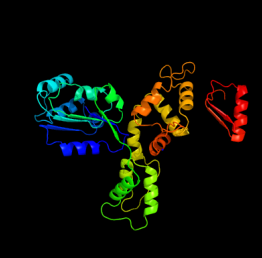

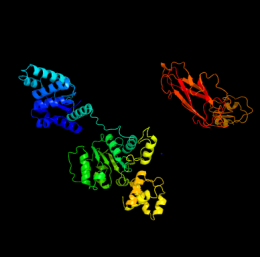


Lcu_2RBY_5g020180_1


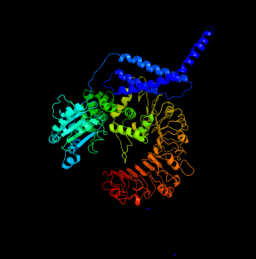


Lcu_2RBY_3g011520_1_


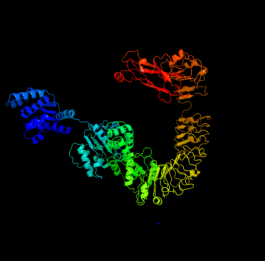


Lcu_2RBY_5g000580_1_


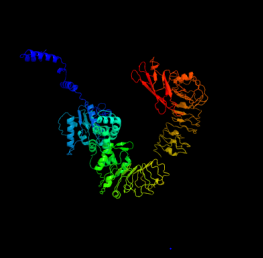


Lcu_2RBY_2g053640_1


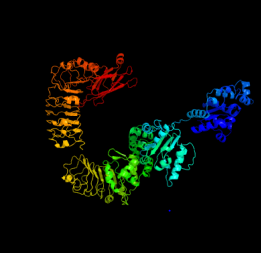


Lcu_2RBY_2g053630_1

Lcu_2RBY_2g031390_1


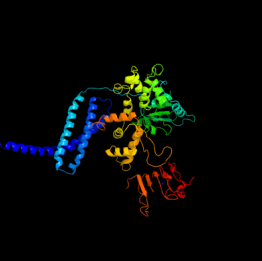


Lcu_2RBY_3g011540_1


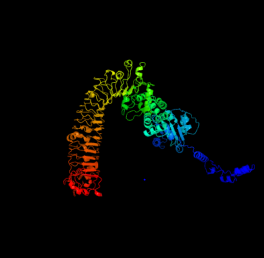


Lcu_2RBY_3g013800_1


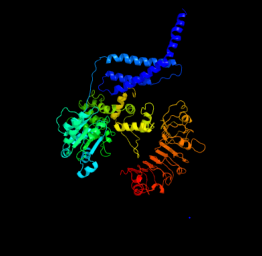


Lcu_2RBY_3g011370_1


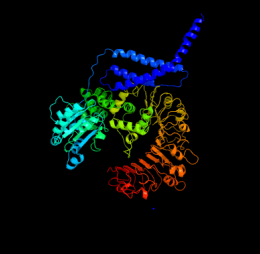


Lcu_2RBY_3g011390_1


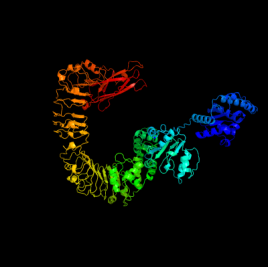


Lcu_2RBY_6g029760_1


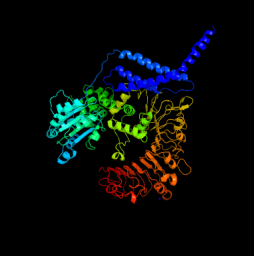


Lcu_2RBY_3g011620_1


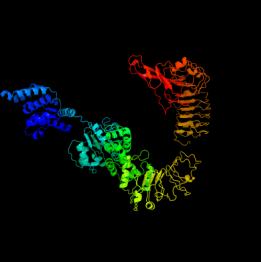


Lcu_2RBY_L012800_1


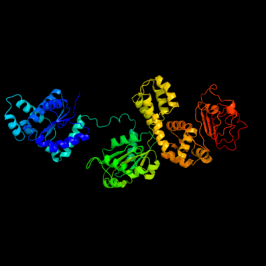


Lcu.2RBY.2g033550.1


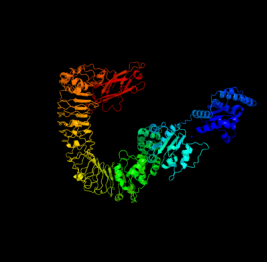


Lcu_2RBY_6g048640_1


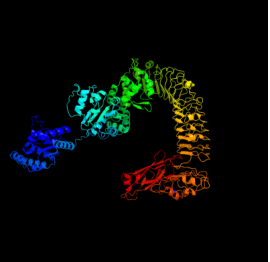


Lcu_2RBY_6g037340_1


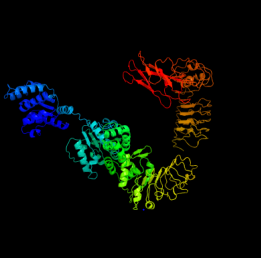


Lcu_2RBY_6g016620_1


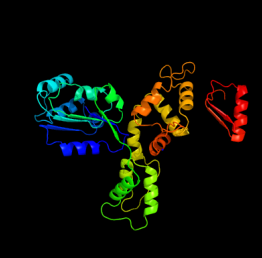

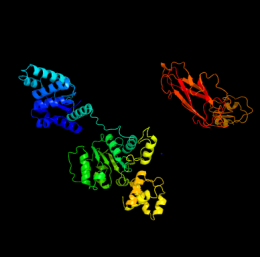


Lcu_2RBY_5g020180_1


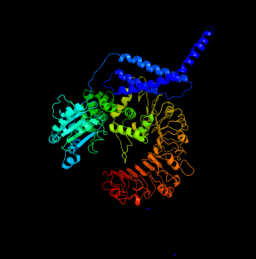


Lcu_2RBY_3g011520_1_


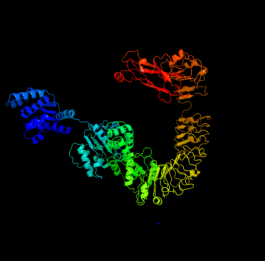


Lcu_2RBY_5g000580_1_


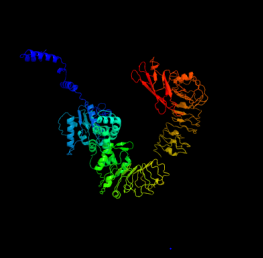


Lcu_2RBY_2g053640_1


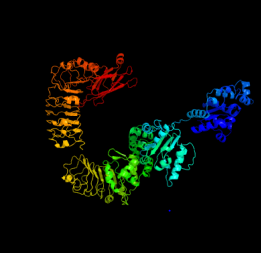


Lcu_2RBY_2g053630_1

Lcu_2RBY_2g031390_1


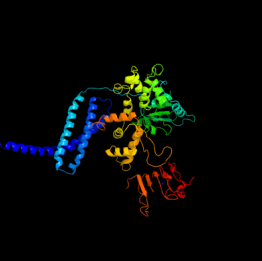


Lcu_2RBY_3g011540_1


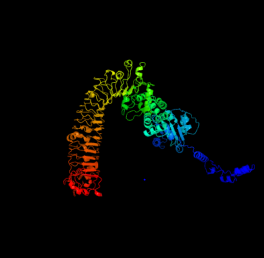


Lcu_2RBY_3g013800_1


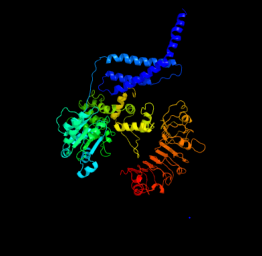


Lcu_2RBY_3g011370_1


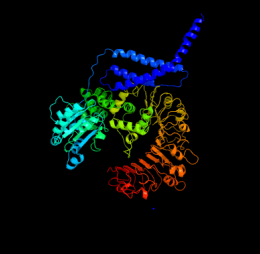


Lcu_2RBY_3g011390_1


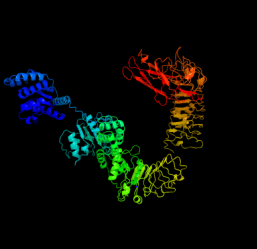


Lcu_2RBY_6g029790_1_


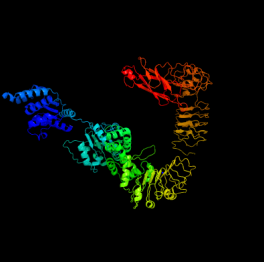


Lcu_2RBY_1g066550_1


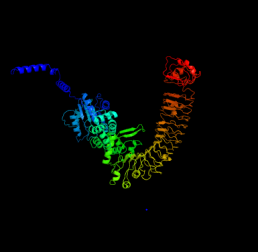


Lcu_2RBY_7g024020_1_

**Supplementary figure 5**. Tertiary structure of lentil R gene predicted using online Phyre 2 software.
